# Biochar-templated surface precipitation and inner-sphere complexation effectively removes arsenic from acid mine drainage

**DOI:** 10.1007/s11356-021-13869-8

**Published:** 2021-04-18

**Authors:** Dongmei Wang, Robert A. Root, Jon Chorover

**Affiliations:** 1 Department of Environmental Science, University of Arizona, 1177 E 4th St, Shantz 429, Tucson, AZ 85721, USA; 2 Department of Environmental Engineering, Southwest Jiaotong University, Chengdu 610031, China

**Keywords:** Biochar, Mine tailings, Arsenic sorption, Iron hydroxide activation, Acidic mine drainage

## Abstract

Treatment of aqueous leachate from acid mine tailings with pristine biochar (BC) resulted in the removal of more than 90% of the dissolved arsenic with an attendant rapid and sustained pH buffering from 3 to 4. Pine forest waste BC was transformed to a highly effective adsorbent for arsenic remediation of acid mine drainage (AMD) because the dissolved iron induced “activation” of BC through accumulation of highly reactive ferric hydroxide surface sites. Physicochemical properties of the BC surface, and molecular mechanisms of Fe, S, and As phase transfer, were investigated using a multi-method, micro-scale approach (SEM, XRD, FTIR, XANES, EXAFS, and STXM). Co-located carbon and iron analysis with STXM indicated preferential iron neo-precipitates at carboxylic BC surface sites. Iron and arsenic X-ray spectroscopy showed an initial precipitation of ferrihydrite on BC, with concurrent adsorption/coprecipitation of arsenate. The molecular mechanism of arsenic removal involved bidentate, binuclear inner-sphere complexation of arsenate at the surfaces of pioneering ferric precipitates. Nucleation and crystal growth of ferrihydrite and goethite were observed after 1 h of reaction. The high sulfate activity in AMD promoted schwertmannite precipitation beginning at 6 h of reaction. At reaction times beyond 6 h, goethite and schwertmannite accumulated at the expense of ferrihydrite. Results indicate that the highly functionalized surface of BC acts as a scaffolding for the precipitation and activation of positively charged ferric hydroxy(sulf)oxide surface sites from iron-rich AMD, which then complex oxyanion arsenate, effectively removing it from porewaters.

## Introduction

Release of toxic metal(loid)s from mine tailings via acid mine drainage (AMD) occurs as a result of dissolution and colloidal dispersion, both of which pose significant risk to human health if introduced to neighboring ecosystems, groundwater, or drinking water supplies. Among the toxic metal(loid)s, arsenic is of particular concern due to its acute toxicity to humans (ATSDR 2011). Arsenic contamination in mineralized regions, and concentrated in tailing dams by beneficiation activity, has resulted in the degradation of proximal surface water, groundwater, and soil, requiring extensive and expensive remedial action (Carlin et al. 2016). Containment is an effective management strategy at point sources of contamination, and successful efforts to amend mine tailings and AMD in-place to decrease risks of human health include excavation, capping, phytostabilization, and permeable reactive barriers (Hammond et al. 2018; Hu et al. 2019; Li et al. 2018; Mendez and Maier 2008; Valentin-Vargas et al. 2013). However, costs and unanticipated knock-on effects of remedial strategies compel further investigations into effective health risk mitigation (Hammond et al. 2020).

Due to its high specific surface area, micro-porosity, and benign character, biochar (BC) or black carbon has been promoted as a potential inexpensive sorbent for remediation of contaminated soil and waste streams (Ibrahim et al. 2016; Liu et al. 2019; Meng et al. 2018; Puga et al. 2016). Biochar is a sustainable product of pyrolyzed waste biomass, including agricultural and forestry residues (Artiola et al. 2012; Kelly et al. 2014; Liang et al. 2016). Wood biochar is dominated by highly aromatic structures with a relatively high specific surface area (between 350 and 400 m^2^ g^−1^) and variable pore volume. Pyrolysis induces dihydroxylation and dehydration reactions that confer alkalinity and precipitation of mineral solids from the biomass including quartz, calcite, and hydroxyapatite (Lehmann and Joseph 2009). Characteristics of the BC surface include reactive (including carboxylic and phenolic) organic functional groups, net negative surface charge, and high interfacial pH, making it a high-affinity absorbent for cations (Beesley et al. 2010; Lehmann and Joseph 2009; Lu et al. 2016). Oxygen-containing BC functional groups can act as Lewis acids that accept electrons during complex formation, generating a pH-dependent charge and even a redox active surface. The basal planes of graphitic micro sites have a high affinity for sorption of transition metal cations via pi-electron bonding (Brennan et al. 2001). The BC interface with aqueous solutions includes surface functional groups, surface radicals, and surface charge, all of which provide important reactive sites for surface complexation reactions (Xiao et al. 2018).

BC has been successfully deployed for the remediation of contaminated military ranges, agricultural soils, and industrial waste streams (Ahmad et al. 2016; Li et al. 2018; Uchimiya et al. 2012). Studies have indicated that transition metal cations (e.g., Cu^2+^, Pb^2+^, Zn^2+^) can be effectively immobilized by adsorption to BC (Beesley et al. 2010; Li et al. 2019). Far fewer studies have focused on the adsorption of metalloid oxyanions because BC exhibits net negative surface charge, and it has been shown to be a low-affinity sorbent for anionic contaminants (Amen et al. 2020). However, results are equivocal. For example, the use of BC as an amendment in an arsenic-contaminated soil–plant system showed diminished arsenic uptake to plants but increased mobilization into soil pore water (Beesley et al. 2013). This was likely due to BC acting as a source of phosphate, which competes with surface sites for arsenate sorption on soil, and preferential uptake of PO_4_^3−^ over AsO_4_^3−^ (Zhao et al. 2019b). Conversely, ternary complex formation, wherein BC serves as an adsorbent for cationic metals or nanoparticulate metal precipitates that could, in turn, present high-affinity complexation sites to adsorptive AsO_4_^3−^ species, could potentially transform the BC surface to an effective adsorbent for AsO_4_^3−^ (Yoon et al. 2017). For example, the use of engineered BC impregnated with magnetite has been shown to effectively remove aqueous arsenate (Zhang et al. 2013; Bakshi et al. 2018), where the mass ratio of Fe to BC ranged from 1:1 to 3:1. Wang et al. (2015) added hematite to BC at a mass fraction of 0.03 Fe and used the composite media for arsenate removal. These techniques utilized the BC as a scaffolding to support added, active ferrous hydroxide particles and associated surface sites for arsenate removal.

In the presence of elevated dissolved ferric and ferrous iron, as occurs in AMD, the adsorption of Fe^3+^ at solid surfaces can initiate surface nucleation and crystal growth of ferric (hydr)oxides. Because iron (hydr)oxides and hydroxysulfates are among the most well-known and effective sorbents for immobilizing inorganic arsenic (Dixit and Hering 2003; Root et al. 2007; Wu et al. 2018), we postulated that biochar could be an effective remedial material for arsenic removal in Fe-bearing AMD waters. Specifically, we hypothesized that it would serve as a templating agent for surface nucleation of reactive ferric solids, and once activated by surface coating, BC would then promote surface complexation and removal of arsenic from the aqueous phase. If effective, such an approach, which would be leveraging the composition of the AMD waste stream itself to make BC a high-affinity adsorbent for arsenic, could be used at relatively low cost and with minimal complexity to remediate arsenic-bearing AMD.

The goal of this work was to evaluate the performance of unaltered BC on arsenic immobilization from AMD generated from metalliferous tailings derived from a sulfide ore deposit. The molecular mechanisms whereby arsenic may be immobilized from AMD waters using unamended BC have not been reported previously, but the presence of dissolved iron originating from mine waters that can subsequently react with BC provides an ideal opportunity to examine the nature and function of iron activation for arsenic removal that would also be applicable to other contaminated sites dominated by pyritic mineralogy.

## Materials and methods

### Preparation and characterization of mine tailing water

Mine tailings were collected from the top 0–30 cm at the Iron King Mine-Humboldt Smelter Site (IKMHSS) Superfund Site, located in central Arizona, USA. The tailings have been well-characterized and contain high levels of arsenic (3 g kg^−1^) and iron (120–150 g kg^−1^) (Hayes et al. 2014; Root et al. 2015). The tailings were field screened to 2.5 cm, homogenized, and further sieved to 2-mm fines. The acid potential (AP) of the tailings was calculated from the mass of pyritic sulfur in the tailings, where total S was determined by total digestion and inductively coupled plasma mass spectrometry (ICP-MS) analysis, and the pyrite fraction determined by synchrotron X-ray absorption near edge structure (XANES) spectroscopy (Solis-Dominguez et al. 2012). Mine tailing pore water (MTW) was extracted by reacting tailing fines with 18.2 MΩ m^−1^ deionized water (Milli-Q, Barnstead) at 25 °C for 24 h in a gentle end-over-end rotator (7 rpm) at a solid to solution mass ratio of 100 g kg^−1^. After the reaction, the mixed solution was centrifuged at 28,000 relative centrifugal force (RCF) for 30 min, pelletizing particles with Stokes’ radii > 0.02 μm. The supernatant MTW solution was aspirated and used in subsequent experiments. Physiochemical parameters and soluble element concentrations were collected in the University of Arizona Laboratory for Emerging Contaminants (ALEC). Measurements of pH and EC were completed with daily-calibrated electrodes (Orion, epoxy semi-micro electrode). Metal(loid) concentrations were determined by ICP-MS (Perkin Elmer DRC-II) after microwave-assisted (Mars6, CEM) nitric acid digestion. Dissolved arsenic was previously determined to be arsenate (As^5+^) by high-pressure liquid chromatography (HPLC, Perkin Elmer Series 200, Hamilton PRX-P column) followed by ICP-MS detection. The dissolved Fe^2+^ was quantified using the ferrozine assay (1% w/v ferrozine in 50% w/v ammonium acetate) with UV detection (Stookey 1970). Analyses of TIC and TOC were by Shimadzu TOC-VCSH analyzer (Columbia, MD). Anion concentrations were measured by ion chromatography (Dionex DX-500, Sunnyvale, CA) with an AS-11 column and a NaOH mobile phase. Detailed properties of the MTW and mine tailings are given (Table S1).

### Preparation and characterization of BC

Biochar was produced from pine forest wastes (including ponderosa pine wood chips, bark, small branches, and pine needles visible to the naked eye) using slow pyrolysis in batch mode with an interparticle temperature of 450 to 500 °C (Artiola et al. 2012). Pyrolyzed particulate samples were sieved to a 1-mm mesh and stored at room temperature. The pH and EC of the BC were measured following 24 h reaction in aqueous suspension (100 g kg^−1^) using 18.2 MΩ m^−1^ deionized (Milli-Q, Barnstead) water. Samples were centrifuged and the aspirated supernatant solution was filtered using a 0.22-μm GHP membrane prior to analysis by ICP-MS and IC as above. Detailed properties of the BC are given in the SI (Table S2). The dissolved organic carbon (DOC) generated by unreacted pine forest waste BC was extracted by separately reacting 1 g of BC sieved to < 250 μm with 20 g of DI water (18.2 MΩ m^−1^) in an end-over-end rotator (7 rpm) for 24 h. The suspension was passed through a 1.2-μm glass fiber filter (GF/C, Whatman) and characterized by STXM and C–NEXAFS (described in “Characterization of the solid phase”).

### Batch kinetic experiments

Kinetic adsorption experiments were performed in triplicate to assess arsenic removal by BC as a function of contact time by adding 0.1 g BC to 10 g of MTW in metal-free 50-mL polypropylene vessels (VWR) at room temperature (25 ± 0.5 °C) in an end-over-end rotator (7 rpm). Sacrificial batch reactors were sampled at pre-determined intervals (0, 15 min, 30 min, 1 h, 2 h, 6 h, 12 h, 24 h, and 48 h). Suspensions were analyzed for pH and then centrifuged at 28,000 RCF. The supernatant was aspirated and filtered through 0.22 μm pore size GHP filters for arsenic and iron analyses. The solids were lyophilized at − 40 °C and 0.130 mbar for subsequent bulk and micro-focused analysis.

To evaluate the effect of pH on arsenic removal, MTW was adjusted to designated pH values between 1.5 and 8 with 0.1 M NaOH and HCl immediately prior to the addition of BC. After 24 h reaction, the suspensions were centrifuged and filtered through 0.22-μm filters for arsenic analysis. The extended buffering capacity of BC was monitored with MTW + BC adjusted to pH 3 with HCl at intervals of 0 h, 0.5 h, 4 h, and 24 h and the serial pH response measured (Fig. S1). Controls with no BC were examined to evaluate the isolated effect of pH on arsenic solubility and complexation in the absence of BC (Fig. S2).

Geochemical modeling was used to calculate aqueous phase iron speciation under the kinetic batch experimental conditions, and to investigate the relation between the observed and thermodynamically predicted mineral phases using Geochemist’s Workbench (GWB) package v 9 with the Lawrence Livermore National Laboratory (LLNL) thermodynamic database thermo.com.v8.r6+ (Bethke 2008; Delany and Lundeen 1990), modified with solubility data for schwertmannite and plumbojarosite (Forray et al. 2014; Majzlan et al. 2006) (Table S3). Activity–activity phase relationship diagrams were calculated using the ACT2 program with activity coefficients calculated with the extended Debye–Huckle B-dot method (Helgeson et al. 1981).

### Characterization of the solid phase

#### X-ray diffraction

Control BC (DI reacted only); MTW-BC at 12, 24, and 48 h; and MTW-BC reacted for 24 h with the pH adjusted from 1.5 to 8 were prepared for powder X-ray diffraction by placing about 50 mg of lyophilized and ground BC between two layers of clear cellulose tape (Scotch Magic^™^). Laue patterns were collected on a large array CCD (MAR3450) detector at the Stanford Synchrotron Radiation Lightsource (SSRL) at beam line 11–3 operating at 500 mA and 12735 eV (*λ* = 0.976 Å). Three exposures of 60 s were collected while the sample was rastered 2 mm in *x* and *y*, normal to the incident beam and averaged. Calibration, geometric corrections, and conversion of the 2-D images to 1-D 2-theta diffractograms were performed with Igor Pro v 8.02 (WaveMetrics, Inc.) using the Nika v1.81 add-on (Ilavsky 2012). The energy was converted to Cu-Kα radiation for comparison with conventional mineral standards using X-Pert HighScore Plus software (PANalytical). Diffractograms were normalized to the maximum broad carbon (002) peak at 3.4 Å. Inorganic crystalline phases were identified with reference minerals from the International Centre for Diffraction Data Powder Diffraction File (ICDD PDF-2) database (ICDD 2005).

#### STXM and C NEXAFS

Scanning transmission X-ray microscopic (STXM) analysis of the lyophilized MTW reacted BC solids was performed at the SM beamline (10ID-1) at the CLS, as above for the DOC. Briefly, about 1 μL of the MTW-BC solids in suspension was deposited onto a 100-nm-thick silicon nitride window (1 × 1 mm) and allowed to air-dry. Stacked maps were collected with a 100-to 150-nm^2^ pixel size at incremental energies across the C K-edge, and difference maps were collected above and below (presence–absence) the Fe and As L-edges, although As was not detected. Collected images were converted to optical density by normalization to an absorbance-free (I_0_) region, and NEXAFS spectra were extracted at each pixel from stacked maps collected across the C K-edge and Fe Ledge and analyzed using principal component and cluster analysis in the MANTiS software package (Lerotic et al. 2014). Incident energy was calibrated with CO_2_ at 290.74 eV. Pseudo-Voigt profile curve-fitting was used to assign peak position (± 0.3 eV) and FWHM using the software package Athena (Ravel and Newville 2005). Peak positions were assigned by comparison with literature-reported values (Lehmann et al. 2005).

#### X-ray absorption spectroscopy (XAS)

Iron and arsenic K-edge XAS were collected with a 100-element Ge array detector, a Si (220) phi = 90 double-crystal monochromator, and 2-mm vertical beam slits at Stanford Synchrotron Radiation Lightsource (SSRL) beam line 11–2. Energy was calibrated for iron and arsenic with the first inflection of the white line absorbance of an iron foil defined as 7112 eV, and the first inflection of the absorbance of a gold foil defined as 11,919 eV, respectively. Lyophilized, unreacted BC and MTW-BC were ground, loaded into aluminum sample holders, sealed with Kapton tape, and placed in an LN_2_ cryostat, with spectra acquired to *k* = 13.5 using 0.35 eV energy steps in the XANES region. All scans (*n* ≥ 4) were averaged using the SIXPACK software package (Webb 2006). Spectra were background subtracted, normalized, and quantitatively analyzed by linear combinations fitting (LCF) using reference minerals collected under similar conditions using the Athena software package (Ravel and Newville 2005). The first derivatives of normalized Fe XANES spectra were fit with a range of 7100–7150 eV. After visual inspection and comparisons of different combinations of reference spectra, the final reference spectra included ferrihydrite, goethite, schwertmannite, and unreacted BC. Arsenic XAS were fit by LCF to reference arsenate sorbed ferric mineral specimens. To further examine the local bonding environment of arsenic at the BC surface, shell-by-shell fits of the *k*^3^ extended X-ray absorption fine structure (EXAFS) by non-linear least squares methods in *k*-space (3–12.5 Å^−1^) were performed with the Artemis software package (Ravel and Newville 2005). Phase-shift and amplitude functions were calculated with the program FEFF8 with self-consistent calculations (Rehr 1993) using atomic clusters taken from the crystal structures of angelellite [Fe^III^_4_(As^V^O_4_)_2_O_3_]. Multiple scattering paths from arsenate tetrahedra (As^V^O_4_), which have been shown to improve the As EXAFS fits, were included (Beaulieu and Savage 2005; Ona-Nguema et al. 2005). The unreacted BC had insufficient arsenic to obtain EXAFS spectra, but quality As XANES data were collected. The shell-by-shell fits were evaluated against the reduced chi and *R*-factor evaluation parameters, where minimization of the fitting parameters indicated a better correlation between the data and the adjusted variables, normalized by the degrees of freedom in the fit.

#### Fourier transform infrared (FTIR) spectroscopy

Lyophilized samples were mixed with KBr at a 1:50 mass ratio, ground by mortar and pestle to homogenize, and pressed into 13-mm-diameter pellets (Spex SamplePrep) for mid-range FTIR spectroscopy (Nicolet 6700 spectrometer, Thermo). Transmission FTIR spectra were collected over the region of 400–4000 cm^−1^ using a CO_2_-free purge gas generator and a deuterated triglycine sulfate (DTGS) detector with 32 interferograms averaged for each spectrum. Data collection and spectral processing, including background subtraction and baseline correction, were performed using the OMNIC program (Thermo Nicolet, Co.).

#### Scanning electron microscopy with energy-dispersive spectroscopy (SEM-EDS)

High-resolution micrographs were collected by field emission scanning electron microscope (FESEM, Hitachi S-4800 Type II) operated at 20 kV with energy-dispersive X-ray spectroscopy (EDS, Oxford) analysis collected at 10 kV. Samples of unreacted BC and MTW-BC (at 48 h) were prepared on carbon tape and imaged under various magnifications at 7.5 mm working distance.

## Results

### Mine tailing water

Mine tailings from the IKMHSS Superfund site have an acid-generating potential (AP) of 1.6 kg H_2_SO_4_ Mg^−1^ (Solis-Dominguez et al. 2012), and produce AMD from the oxidization of sulfides and hydrolysis of Fe^3+^_(*aq*)_. Water extracted from mine tailings was acidic and had elevated conductivity (pH = 3.2 (± 0.1), EC = 7.22 (± 0.01) mS cm^−1^) (Table S1). The water-soluble inorganic and organic carbon values were (48.7 (± 1.1) mg kg^−1^ and 59.9 (± 1.4) mg kg^−1^, respectively). Dissolved arsenic in MTW was 7.7 μmol kg^−1^ and dissolved iron was 24 mmol kg^−1^. The oxidation state of arsenic in solution was As^V^, which, at pH 3.2, would be dominated by the oxyanion H_2_AsO_4_^−^ (pKa = 2.19). Dissolved iron was dominantly ferric (Fe^3+^ = Σ(FeOH^2+^, Fe(OH)^2+^, Fe^3+^) with a small fraction of ferrous (6.4% of total Fe) (Table S1). Dissolved sulfur was dominantly sulfate, with total S concentration = 226 mmol kg^−1^.

### Arsenic adsorption kinetics

**Fig. 1:**
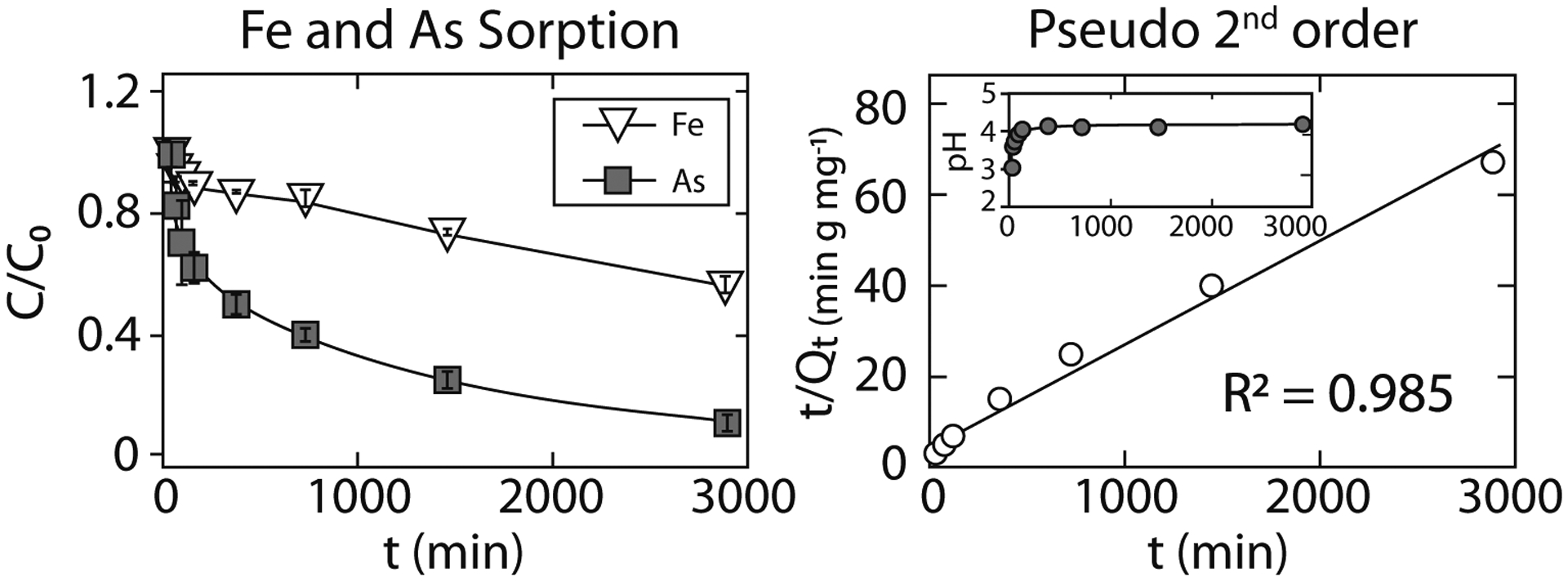
Iron (open triangles) and arsenic (filled squares) removal from solution (left panel), with arsenic loss fit to pseudo-second-order rate expression (right panel), and reaction pH shown in inset. Error bars, generally smaller than the symbols, represent the standard deviation of triplicate samples. *C/C*_*0*_ is the fraction of Fe or As in solution, *t*/*Q*_*t*_ (min mg g^−1^) is the quotient of reaction time *t* (min) and adsorbate (As) concentration at time *t* (Jin et al. 2020)

The arsenic removal rate was analyzed by monitoring the solution chemistry of the MTW-BC reaction from 15 min to 48 h (Fig. 1). As the reaction progressed, pH rapidly increased from 3.2 to 4.0 and reached an apparent equilibrium within 2 h. Dissolved arsenic ([As]_0_ = 580 μg kg^−1^) was removed from solution at each time step to the end of the experiment. At 48 h, the concentration of arsenic in solution approached the limit of detection (Fig. 1). The adsorbate concentration (*Q*, mg g^−1^) as a function of time (*t*) (min) was well described by a pseudo-second-order model (*R*^2^ = 0.985) (Fig. 1), and less well described by either a first-order expression (*R*^2^ = 0.833) or Elovich equation (*R*^2^ = 0.826) (Fig. S3), consistent with arsenic removal to a reactant that is not present in excess.

### Characterization of the reacted biochar

**Fig. 2:**
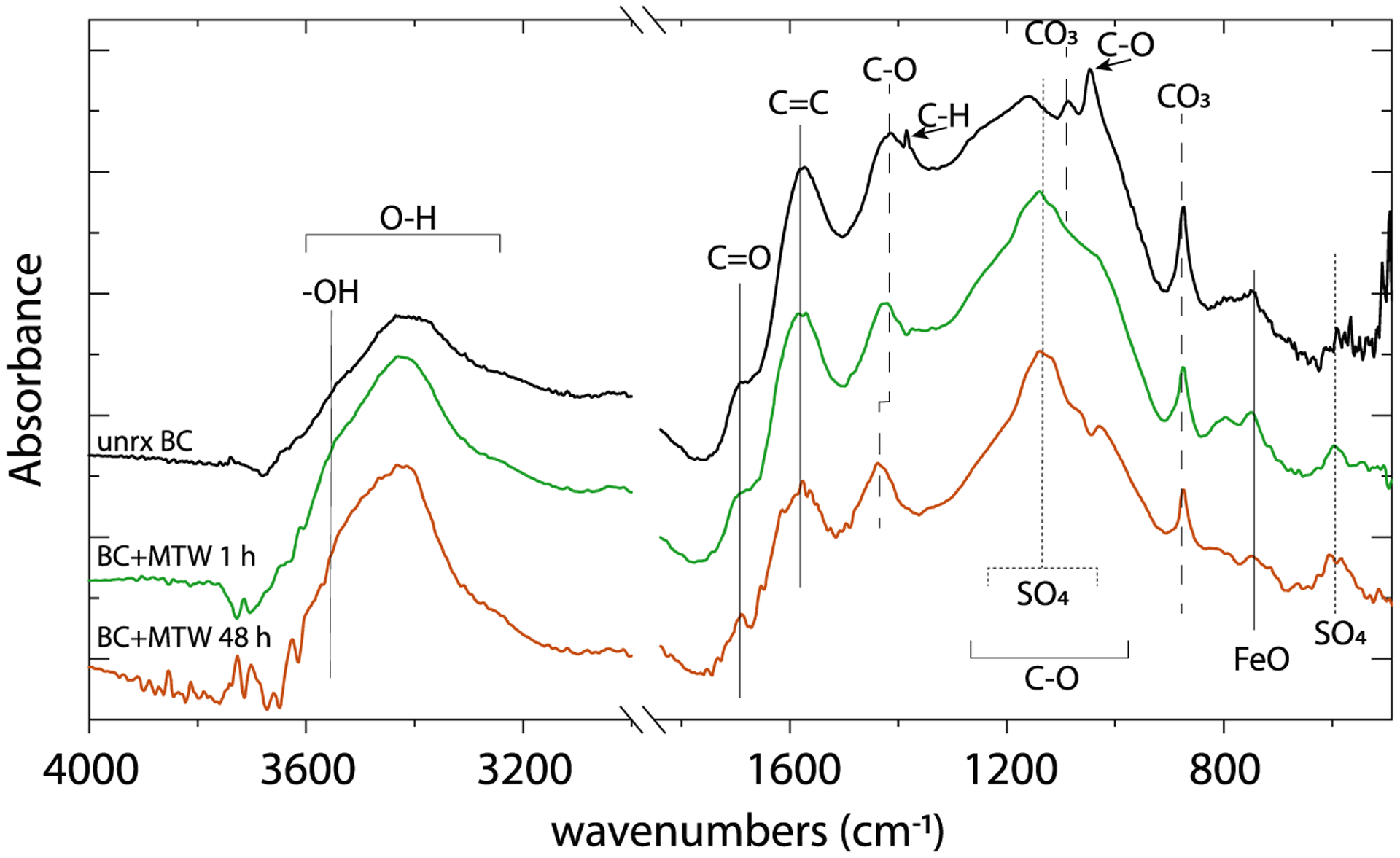
FTIR spectra of pine forest waste biochar before (black) and after reaction with MTW at 1 h (green) and 48 h (brown). Spectra are dominated by organic aromatic and aliphatic vibrations. Noteworthy changes include reduced CO_3_ vibrational bands, increased broad SO_4_ absorbance (1100–1200 cm^−1^), and increased free hydroxyl groups (3550–3600 cm^−1^)

Functional groups and treatment effects were identified for the unreacted BC and MTW-reacted BC for 1 h and at 48 h by FTIR spectroscopy (Fig. 2). FTIR spectra evolved with reaction time. A broad band centered at *ca*. 3400 cm^−1^, representing O–H stretching, indicated abundant oxygen-containing functional groups in the BC (Yuan et al. 2011). After reaction with MTW, the intensity of the broad 3300–2500 cm^−1^ hydroxyl stretching band was reduced, and an increase centered at 3500 cm^−1^ was noted. The small feature at 1690 cm^−1^ increased with the MTW reaction time, attributed to C=O stretching. The C=C stretch from poly-aromatics at 1576 cm^−1^ was generally unchanged pre- and post-MTW reaction, indicating a stable aromatic biochar backbone structure. However, the band centered at 1437 to 1414 cm^−1^, assigned to C–O stretching, shifts to higher frequency with reaction time in MTW, an observation that can be attributed to increased H-bonding in the acidic environment. The peaks at 1432, 874, and 748 cm^−1^, from the asymmetric CO_3_ stretch for calcite, were diminished in the MTW-BC, as expected by calcite dissolution induced by the acidic pH of the MTW. A small peak in the fingerprint region at 1050 cm^−1^, attributed to C–O stretching, is removed after reaction with MTW, and a small, broad peak emerged upon MTW reaction at 605 cm^−1^, which could be attributed to Fe–O stretching. Along with the diminished calcite peaks, the largest difference between the BC control and MTW-BC was the wide feature from approximately at 1100–1200 cm^−1^, a region generally assigned to sulfate, which was strengthened with increasing MTW-BC reaction time. The difference between the unreacted BC and MTW-BC in this region was consistent with sorbed SO_4_^2−^ or precipitation of gypsum and/or schwertmannite (Fig. S4).

**Fig. 3:**
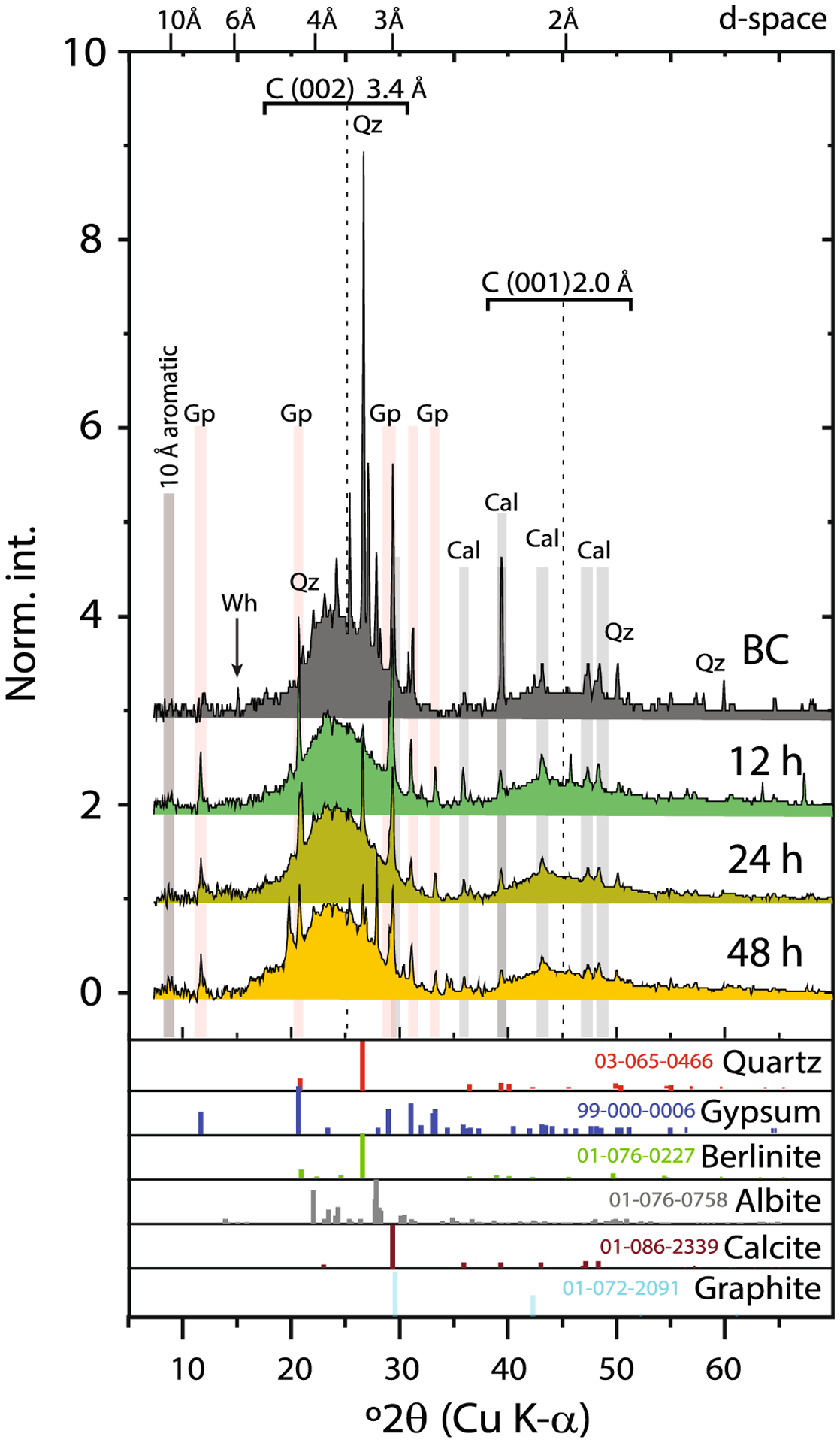
X-ray diffraction patterns for the unreacted and MTW-reacted biochar. Diffractograms of unreacted BC and MTW-BC for 12, 24, and 48 h, normalized to the maximum carbon (002) 3.4 Å feature. The *x*-axis shows °2*θ* (*λ* = Cu); the top *x*-axis shows d-spacing (Å). Broad features indicative of turbostratic carbon are noted at 3.4 and 2.0 Å. Cal = calcite, Qz = quartz, Gp = gypsum, and Wh = whewellite. The 10 Å aromatic, an undifferentiated peak characteristic of aromatic carbon rings, increases relatively with reaction time. Standard minerals and the ICCD PDF codes are shown for reference

The X-ray diffractograms of the BC and MTW-BC (48 h) indicate the presence of inorganic quartz at 3.34 and 4.26 Å (011, 100) in all BC samples (Fig. 3). Calcite was confirmed for reactions carried out at pH > 3.5 by Bragg reflections at *d*-spacings of 3.86, 3.03, 2.50, 2.28, 2.09, 1.91, and 1.87 Å (Fig. S5, Table 1). The intense sharp peak at 3.03 Å indicated that calcite was well crystallized. All biochar samples had broad features centered at 3.4 Å (graphite [002]) and 2.0 Å (graphite [001]), characteristic of turbostratic crystalline carbon. Identified solids in the unreacted BC were quartz (SiO_2_), calcite (CaCO_3_), graphite (C), albite (NaAlSi_3_O_8_), whewellite (CaC_2_O_4_·H_2_O), and a peak associated with aromatic rings at 10 Å (Table 1).

However, the mineral assemblage in BC was significantly altered following reaction with MTW. Peaks from calcite weakened or disappeared, due to the instability of carbonates in the low-pH MTW. Similarly, calcium oxalate (whewellite) peaks diminished or disappeared in the acid MTW, indicating dissolution of these solids. Additionally, new peaks (4.27 Å, 3.67 Å, 3.53 Å, 3.24 Å, 2.69 Å) were observed in MTW-BC indicating precipitation of gypsum (CaSO_4_·2H_2_O). Interestingly, no ferric solids were observed in the XRD patterns for MTW-BC. Since other data sources (discussed below) indicate ferric solid precipitation, this XRD result is suggestive of low crystallinity or concentrations below detection of the neo-formed precipitates.

### Molecular speciation of carbon, iron, and arsenic

**Fig. 4:**
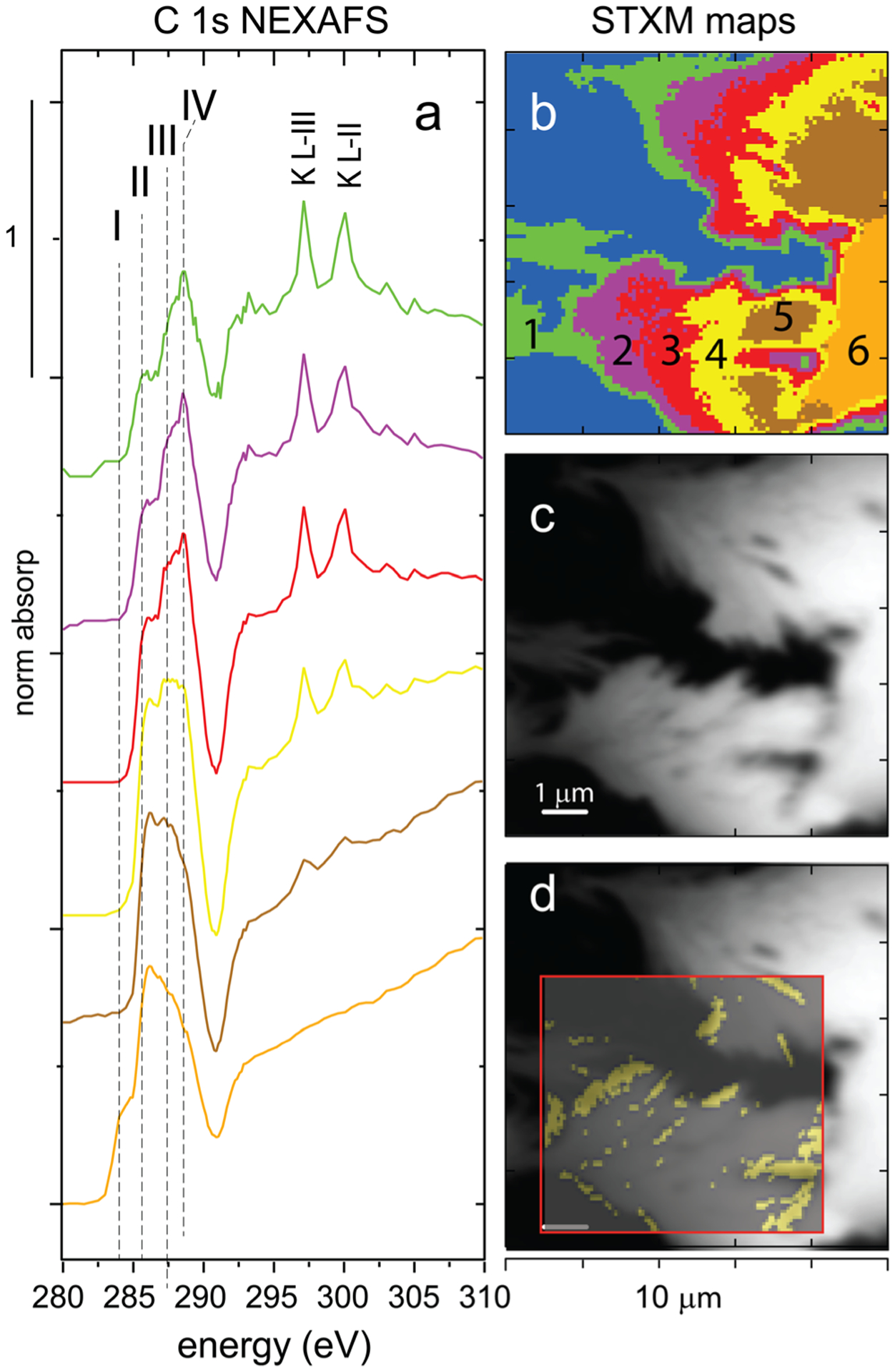
Mine tailing water reacted biochar (MTW-BC) carbon 1s NEXAFS and STXM stacked maps. **a** C-NEXAFS spectra of PCA-determined components; **b** spatial distribution of components in **a**; **c** STXM submicron resolution optical density image; **d** Fe-rich regions overlaying the C map. NEXAFS features I–IV highlight specific carbon components (Lehmann et al. 2005). The peaks at 297.4 and 299.7 are the L-III and L-II peaks of potassium. Please refer to the electronic version for color

The carbon character of MTW-BC (48 h) particles was analyzed by scanning transmission x-ray microscopy (STXM) and carbon 1s near-edge x-ray absorption fine structure (C-NEXAFS) spectroscopy. Principal component analysis (PCA) from the stacked maps collected across the C 1s edge identified seven components, inclusive of the I_0_ carbon-free regions (Fig. 4). Normalized NEXAFS spectra were extracted for each PCA determined region (Fig. 4a), and per pixel analysis of the PCA regions indicated 31.4% of the image corresponded to the I_0_ background, and components 1–6 accounted for 13.2, 10.8, 13.2, 10.8, 14.0, and 6.6% of the pixels in the map, respectively (Fig. 4b). Select C 1s peak features are indicated with dashed vertical lines and labeled with roman numerals (Fig. 4a). The small peak at (I) 284 eV is attributed to a quinone-C surface group 1 s-π*(C=O); (II) the large peak at 285.6 eV is from aromatic-C ring 1 s-π*(C=C); (III) 287.4 is aliphatic-C 1 s-σ*(3p_C-H_); and (IV) 288.5 eV assigned to carboxylic-C 1 s-π*(C=O) (Solomon et al. 2012). Maps in Fig. 4b–d are 10 μm square with a 100-nm^2^ pixel size; the iron map inset was 75 μm^2^ with a 150-nm^2^ pixel size. The regions with high iron correspond to PCA regions 2 and 3, which have C spectra that show strong carboxylic character (288.5 eV). The PCA regions 4, 5, and 6 are not correlated with iron and have C character similar to unreacted BC DOM (Fig. S6).

**Fig. 5:**
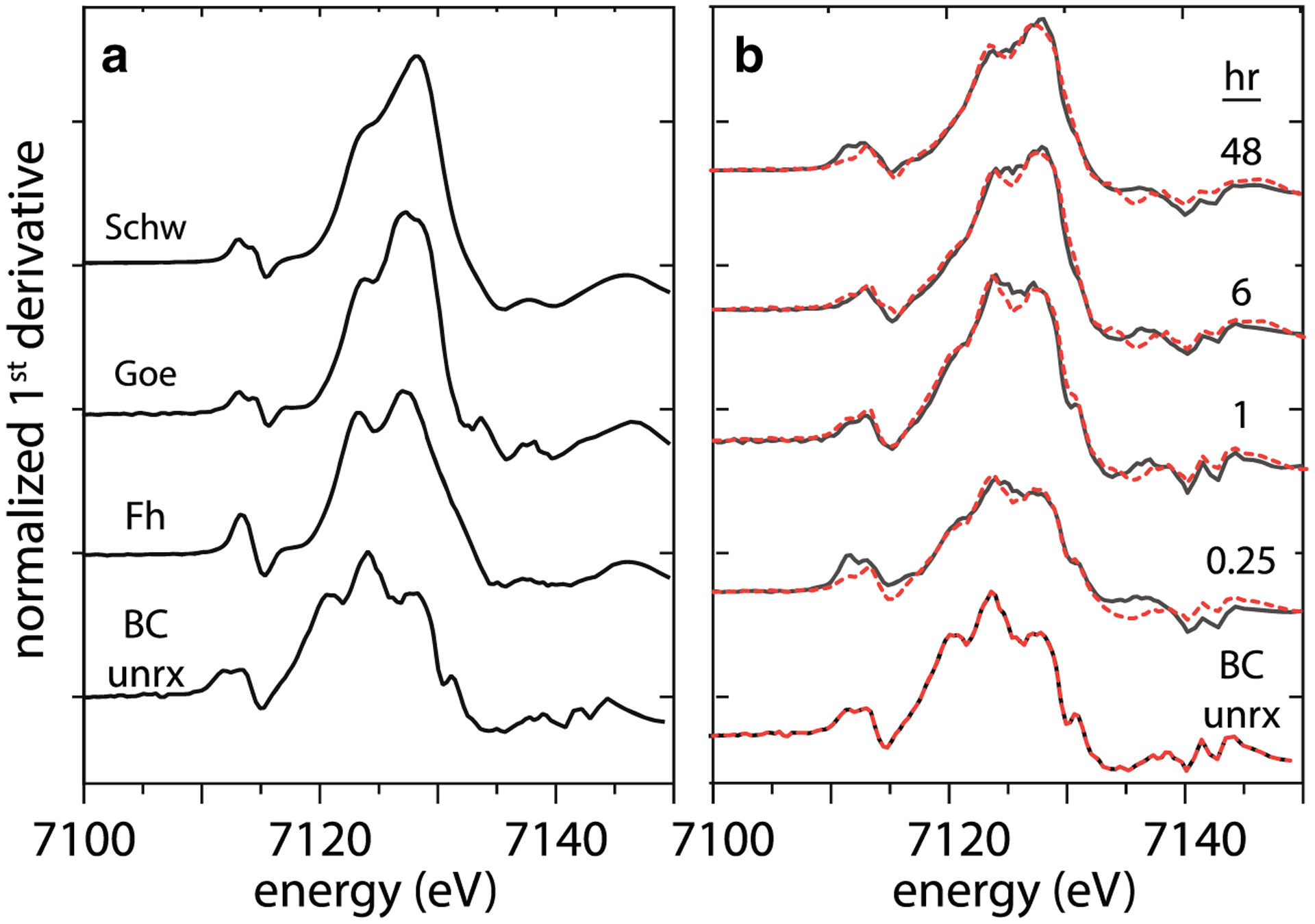
Normalized first derivative iron K-α XANES. **a** Reference spectra. **b** MTW-BC reacted sample spectra. Data shown with black lines, linear combination fits (LCF) to reference spectra shown in dashed red lines, LCF fits given in Table 2

The solid-phase iron species were investigated by Fe Kα XANES in the unreacted BC and in the MTW-BC samples collected at 15 min, 1 h, 6 h, and 48 h (Fig. 5), with fractional components determined by linear combination fitting to the first derivative of XANES spectra (Table 2). The unreacted BC ([Fe]_0_ = 821 mg kg^−1^) XANES spectrum was used as the endmember component for fitting the reacted BC. Iron in MTW-BC reacted for 15 min was mostly that in unreacted BC (70%), but even at this first time point, ferrihydrite [FeOOH-Fe_10_O_14_(OH)_2_] accounted for almost a third of the total iron (30%). As the reaction progressed, the relative fraction of iron from unreacted BC decreased while the secondary iron (hydr)oxide component increased in prevalence with a concurrent change in speciation. Whereas ferrihydrite was identified at each time step (15 min to 48 h), schwertmannite (Fe_8_O_8_(OH)_6_SO_4_) was identified when the reaction time reached 6 h and it remained at 48 h. Goethite (α-FeOOH) was fit to the spectra when the reaction time reached 1 h, and the goethite fractional contribution to the Fe XANES increased from 1 to 6 h and remained about one third of the iron signal from 6 to 48 h.

**Fig. 6:**
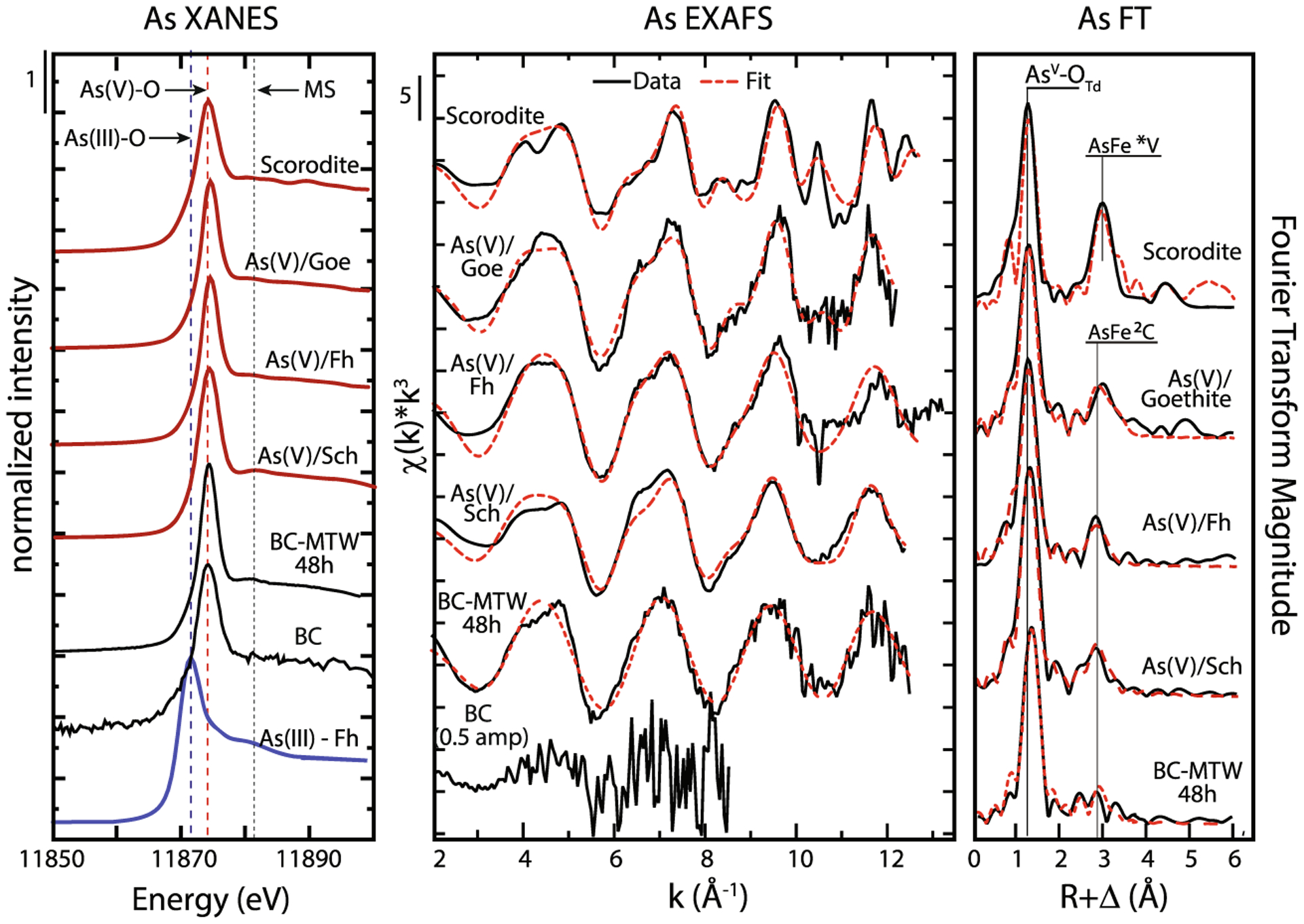
Arsenic K-edge XAS of BC and MTW-BC (48 h) and reference compounds: XANES (left), *k*^3^-weighted EXAFS (center), and Fourier transformed radial distribution function (right). Data shown with black lines, fits to data shown in dashed red lines. Model compounds include scorodite, As^v^ on goethite, As^v^ on ferrihydrite, As^v^ coprecipitated with schwertmannite, and As^III^ on ferrihydrite. Arsenic speciation is indicated in the XANES panel with vertical lines corresponding to the reference minerals: arsenite as As^(III)^-Fh is the blue dashed line, and arsenate as As^(V)^-Fh is the red dashed line. EXAFS fit details in Table S4. See electronic version for color

The oxidation state and speciation of arsenic in BC and MTW-BC were measured with X-ray absorption spectroscopy (XAS). XANES indicated the oxidation state was all pentavalent (As^V^, i.e., arsenate), with an absence of either arsenite, arsenic sulfide, or thiol complexes (Fig. 6). The initial BC had trace arsenic content (0.60 ± 0.10 mg kg^−1^), which was also determined by XANES to be arsenate. Arsenic EXAFS of MTW-BC were examined by LCF and showed good correlation (*R*-factor 0.0999) with a combination of As^V^ sorbed goethite, ferrihydrite, and schwertmannite (Fig. S7). However, the coordination of arsenate tetrahedra at ferric hydroxide octahedra surface sites are very similar across these three ferric solids, and hence, the EXAFS are very similar as well. Therefore, while we can conclude that arsenate is adsorbed at Fe^III^ octahedral sites of mineral plaques formed on the BC surface, LCF has limited applicability for quantitatively deconvolving the relative contribution of these three ferric solids to that arsenate adsorption. The BC, with low arsenic, was sufficient for arsenate speciation by XANES, but the EXAFS signal to noise was too poor for shell-by-shell fitting. Conversely, the MTW-BC produced As Kα EXAFS that were fit with a first coordination shell of 4.0 oxygen atoms at a distance of 1.69 Å, characteristic of the As^V^-O_4_ tetrahedra and in agreement with the assignment of arsenate by XANES.

As EXAFS fit details are provided in Table S4. The arsenic second shell was best fit with 1.2 Fe backscatters at 3.37 Å, a distance longer than model studies with As^V^ coprecipitated with ferrihydrite (Gao et al. 2013), but consistent with bidentate–binuclear corner linkage (^2^C) of As^V^–Fe^III^ at a distance of ~ 3.3 Å, which corresponds to sharing of two arsenate apical oxygen atoms with adjacent edge-sharing Fe(O,OH)_6_ octahedra (Farquhar et al. 2002; Maillot et al. 2013; Manning et al. 2002). This is a likely coordination environment based on the high iron content (1.3 g kg^−1^) and the strong affinity of As^V^ for ferric (hydr)oxides (Dixit and Hering 2003). The ^2^C coordination has previously been shown to be the dominant mode of arsenate tetrahedra adsorption to octahedra of iron (hydr)oxides including goethite, lepidocrocite, hematite, hydrous ferric oxide (HFO), ferrihydrite, schwertmannite, and arsenic-loaded jarosite (Paktunc and Dutrizac 2003; Root et al. 2007; Savage et al. 2000; Sherman and Randal 2003). The MTW-BC As–Fe distance at 3.37 Å was similar to the distance observed for amorphous ferric arsenate (3.36 Å) and scorodite (3.37 Å) (Foster et al. 1998), consistent with a ferric arsenate surface complex (Voegelin et al. 2007). However, the coordination number of 1.2, which was lower than the expected 2, indicated some arsenic was not in ^2^C coordination. A possible sink for arsenic is the mineral schwertmannite, which has been shown to form ^2^C surface complexes as well as structural incorporation. Arsenate oxyanions can displace hydrogen-bonded sulfate ions in the schwertmannite tunnel structure (accepting an akaganeite structure), which forms an outer-sphere complex that is not probed with EXAFS, and would result in a lower coordination number from EXAFS fits (Burton et al. 2009; Maillot et al. 2013), consistent with the results of the present work.

### Morphological alteration of BC

**Fig. 7:**
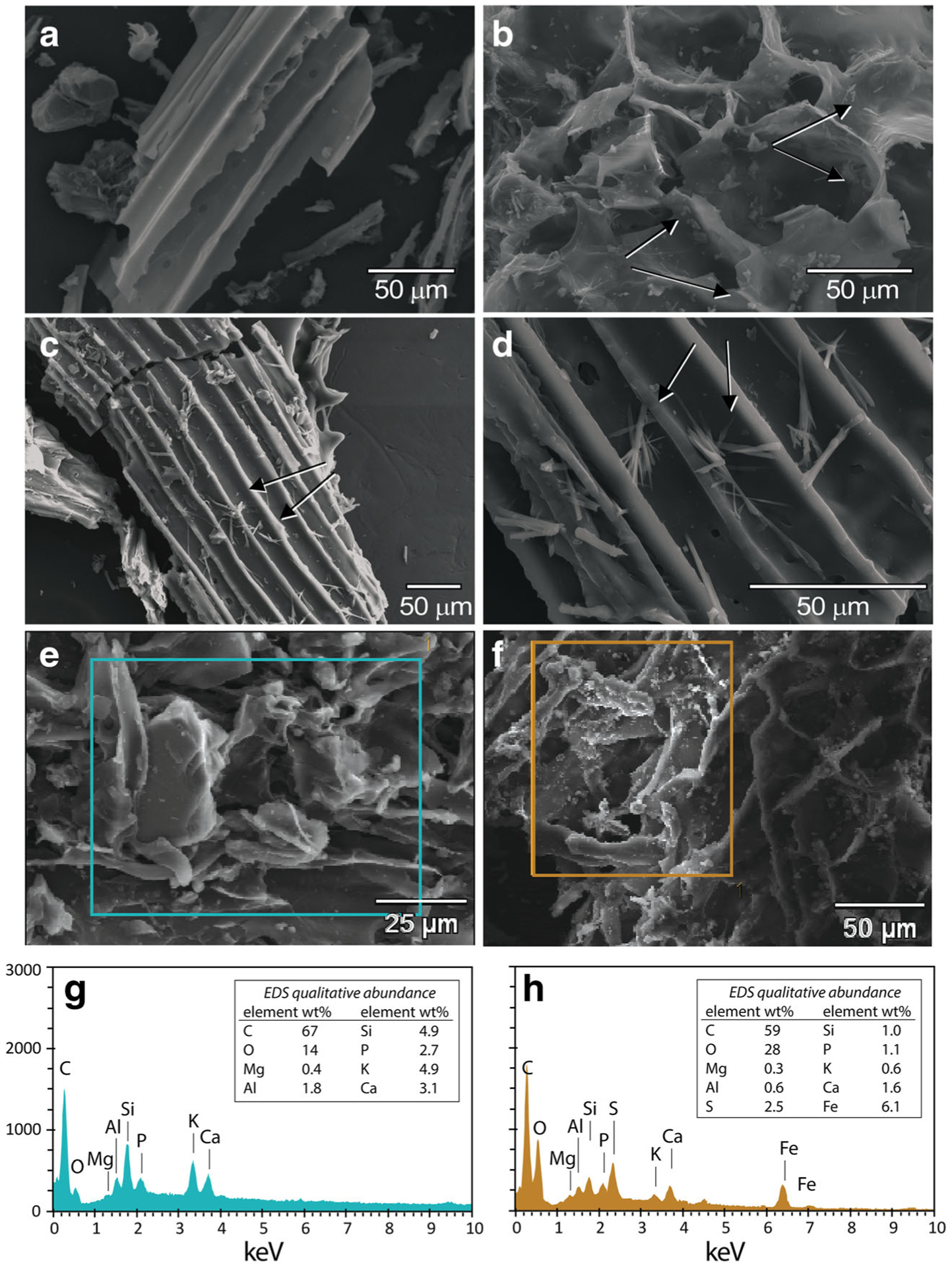
SEM images of unreacted BC and MTW-BC-48 h. **a** BC; **b** MTW-BC with acicular crystals and spherical neo-aggregates; **c** and **d** MTW-BC with acicular neo-precipitates; **e** BC; **f** MTW-BC 48 h with surface coatings; **g** EDS of box in **e**; **h** EDS of box in **f**. Scale given in micrograph panel; collected at 20 kV and 7.5 mm working distance; EDS collected at 10 kV

Micrographs show BC and MTW-BC (48 h) at different magnifications and on different BC particles (Fig. 7a–f). The unreacted BC shows no detectable secondary mineral phases (Fig. 7a). The MTW-BC (48 h) showed a strong emergent signal from S and Fe in association with the precipitated particles on the surface, consistent with the Fe XANES (Fig. 7e, f). Spherical neo-aggregates were observed and could indicate schwertmannite or goethite (Fig. 7b, f). The acicular crystals, consistent with either goethite or gypsum, were observed in the MTW-BC (Fig. 7c–d). Micrographs of BC and MTW-BC show surface texture (Fig. 7 e, f). Carbon and oxygen were the two main elemental components in the BC, with Si, K, and Ca contributing to the X-ray spectrum collected by EDS (Fig. 7g), the MTW-BC(48 h) shows peaks of S and Fe that are not present in the unreacted BC.

## Discussion

### Effect of mine tailing water chemistry on biochar activation

The oxic MTW, with low pH and high dissolved solids inclusive of elevated Fe^3+^_*(aq)*_ and As^5+^_*(aq)*_, is representative of AMD that ponds and seeps from sulfidic mine tailings. Dissolved arsenic in the prepared MTW from IKMHSS mine tailings far exceeds the recommended upper limit of 10 μg L^−1^ for potable water (WHO 2017), and in the absence of acid neutralization and a stable sorbent, Fe^3+^ and As^5+^ in AMD is labile and can contribute to groundwater contamination and offsite ecosystem degradation. The abundant carbonate, oxalate, and carboxyl groups including calcite, whewellite, and R–COO^−^ functional sites in BC provide a strong proton buffering capacity (Yuan et al. 2011), making BC a beneficial amendment under acidic conditions including AMD. It was observed that calcite and whewellite disappeared or weakened in FTIR spectra and XRD diffractograms upon reaction with MTW (Figs. 2 and 3), evidence of BC contribution to mine tailing buffering during reaction. The pH buffering capacity of BC is evidenced by the fact that MTW pH was increased and maintained from about 3 to 4 (Fig. S1) despite ongoing Fe^III^ hydrolysis reactions. The pKa values of carbonic acid, oxalic acid, and common carboxylic acids are 6.4, 4.3 (pKa_2_, whereas pKa_1_ = 1.25), and ca. 4.5, respectively, and the abundant acid-base reactions of BC act to maintain a pH of just above 4 in the AMD modeled here. Whereas dissolved ferric iron is stable under strongly acidic conditions, it is insoluble under the mildly acid pH environment resulting from reaction with alkaline BC. The acid-neutralizing capacity of BC promotes Fe^3+^ hydrolysis and ferric (hydr)oxide precipitation, and the BC surface provides a high interfacial area for mediating nucleation and growth of the surface precipitates. Upon formation, ferric (hydr)oxides on the BC surface provide high-affinity surface sites (octahedral Fe^III^–OH groups) for inner-sphere, bidentate, binuclear complexation of dissolved arsenate, thereby limiting contaminant flux.

### Iron oxide–activated biochar and arsenic removal mechanisms

Dissolved iron in MTW, [Fe] = 1340 ± 50 mg kg^−1^, far exceeded the concentration of iron solubilized from the BC itself; water-soluble BC [Fe] was 0.006 mg kg^−1^. The BC was reacted at 1:100 solid to solution with MTW, where dissolved iron from MTW was more than six orders of magnitude greater than that from the BC alone, making any Fe released from the biochar negligible. Effectively, all of the dissolved and reactive iron was from the MTW. The relative decrease in the BC contribution to the Fe XANES spectra is attributed to neoformed Fe^III^ surface precipitates, and we assume there was no change in the original BC iron species. At the end of the experiment (48 h), the initial iron and neo-precipitate iron, determined by XANES, were approximately equivalent in mass, indicating about 800 mg of newly precipitated ferric hydroxy(sulf)oxide partitioned to each kilogram of BC, which became progressively available for surface complexation with adsorptive arsenic. Control treatments showed that MTW did not produce ferric precipitates within 48 h in the absence of BC, and dissolved arsenate (54.7 μg/L HAsNa_2_O_4_·7H_2_O) added to BC in the absence of MTW was not removed at either pH 3 or 5.8 (Fig. S8), which brackets the pH values observed in the MTW-BC experiments. Hence, the arsenic remediation effect was the result MTW-BC interaction.

When MTW was added to BC, iron partitioning to the solid phase was observed within the first 15 min (Fig. 5). Dissolved iron and arsenic partitioned to the solid phase at an Fe:As mol ratio of about 525 (Fig. S9), and the sequestered arsenic surface coverage was far below the capacity of the phases observed (goethite < schwertmannite < ferrihydrite) (Asta et al. 2009; Burton et al. 2009; Wilkie and Hering 1996). This indicates that MTW-activated BC has additional unused capacity for arsenic removal, and that the surface loading capacity of iron-activated BC should be further investigated. Ferrihydrite and schwertmannite have been shown to have higher arsenate adsorption capacities than goethite does, but the short-range-ordered phases are considered lessrobust sequestration sitesdue to their meta-stability, and tendency to undergo Ostwald ripening to more thermodynamically stable mineral species like goethite (Das et al. 2013). Because arsenic was not removed by unactivated BC, components of mine tailing water, specifically the high dissolved iron, play a vital role in the ability of BC to remove arsenic from water, and activated BC can be an effective remediation media where dissolved iron concentrations greatly exceed those of dissolved arsenic. This condition is met by many AMD systems.

Despite the fact that ferric adsorption sites were apparently in excess of adsorptive arsenic, kinetic data were best fit to a pseudo-second-order model, suggesting adsorption site limitations. This can be attributed to the fact that the kinetically limiting step for arsenic removal was the precursor reaction of ferric mineral plaque formation, which, as indicated by C NEXAFS and STXM results, was nucleated at carboxyl-C-enriched sites in BC (Fig. 3). Surface carboxyl groups in BC are known to have an affinity for polyvalent metal cations and can stabilize metals at surface functional groups (Uchimiya et al. 2012). Additionally, because biochar is redox-active due to its quinone and aromatic structures, it may have the potential to catalyze abiotic surface redox reactions and facilitate ferric hydroxy(sulf)oxide formation from ferrous iron by Fe^II^ oxidation at electron-accepting surfaces (Cataldo et al. 2018; Klüpfel et al. 2014; Yu et al. 2015; Zhao et al. 2019b). The redox active surface did not, however, promote any reduction of arsenate to the more toxic and labile arsenite (Fig. 6). Whereas the MTW solution in the current study was dominated by Fe^III^ aqueous species and the possible oxidation of ferrous iron by BC was not specifically probed, it has been reported previously that Fe^2+^_(aq)_ can form complexes at anionic –C(=O)– surface sites of BC, where electron transfer to the BC produces an insoluble ferric crust (Kappler et al. 2014). Whereas native BC, comprising graphitic sheets and negatively charged functional groups, eschews oxyanion interaction (Lehmann and Joseph 2009; Uchimiya et al. 2012), the ferric-oxide-activated-BC surface complexes promote oxyanion arsenate surface complexation and coprecipitation at iron-activated nucleation sites (Fig. 5). The inner-sphere surface complexation of arsenate by iron oxide is proposed to be a two-step process of initial monodentate ligand exchange with hydroxide at an apical oxygen site of an Fe^III^ octahedron, followed by a second similar ligand exchange reaction on a neighboring Fe^III^ octahedron, resulting in a bidentate, binuclear surface complex (Grossl et al. 1997). The high stability of the bidentate surface complex stabilizes the arsenate against desorption, and it should persist under environmental conditions that favor ferric solids.

**Fig. 8:**
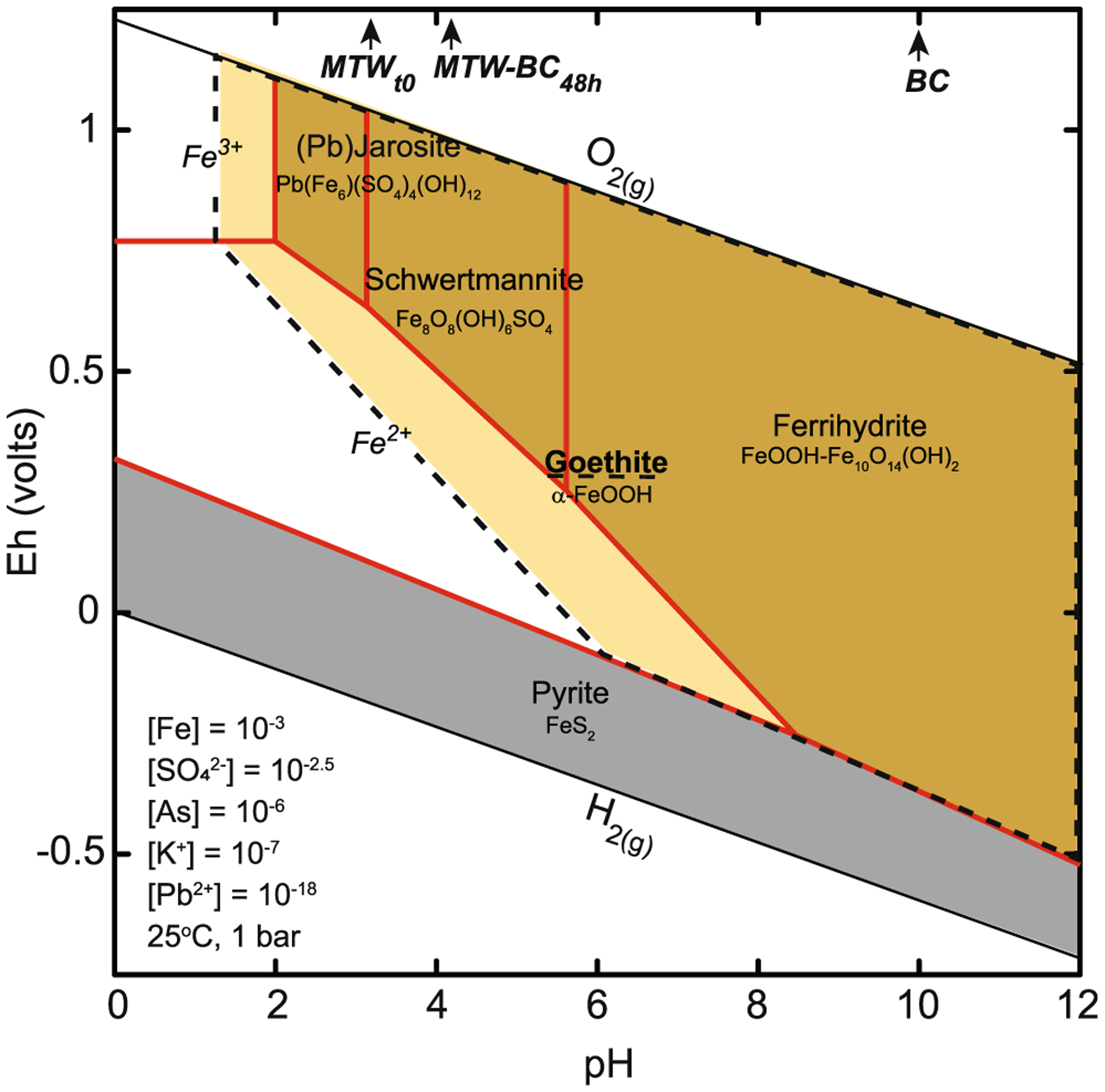
Predominance diagram showing aqueous (unshaded) and solid (shaded) iron species calculated as a function of *E*_h_ and pH at 25 °C and 1 atm with [Fe]_*total*_ = 10^−3^, [SO_4_^2−^] = 10^−2.5^, [As] = 10^−6^, [K^+^] = 10^−7^, and [Pb^2+^] = 10^−18^ using the LLNL thermodynamic database. Modeled solid lines bound the metastable solid phases when goethite is suppressed. The hatched area bounds the stability field of goethite when it is included in the model. The pH of MTW, MTW-BC at 48 h, and BC are indicated at the top *x*-axis with arrows

The dissolved iron species in the MTW was mostly (~94%) ferric, which upon sorption on BC precipitated as ferrihydrite, schwertmannite, and/or goethite. When goethite was suppressed in the thermodynamic model to simulate the kinetic retardation of its precipitation (i.e., < 1 h reaction), the meta-stability fields of ferrihydrite and schwertmannite are shown to have a pH-dependent stability boundary at pH 5.8, with ferrihydrite stable at higher pH and schwertmannite stable at lower pH (Fig. 8). The pH at the end of the experiment was above 4, and at equilibrium under these acid conditions, ferrihydrite is expected to transform to goethite or schwertmannite (Jönsson et al. 2005; Yee et al. 2006). During the initial stages of the reaction, from 15 min to 1 h, the fraction of ferrihydrite was generally unchanged, but at 6 h, schwertmannite was observed as the fraction of ferrihydrite decreased. It is not clear if ferrihydrite was transforming to schwertmannite or if schwertmannite was co-precipitating with ferrihydrite. It can be inferred that while the relative contribution of the iron present in the unreacted BC to the iron XANES signal decreased, the abundance did not change; rather, the contribution from neo-ferric precipitates increased in relative contribution to the spectra. Therefore, about 60% of their on XANES signal was from neo-ferric hydroxy(sulf)oxides after 6 h. At 6 h, ferrihydrite decreased in relative predominance and goethite and schwertmannite appeared. The transformation of ferrihydrite to goethite did not inhibit the rate of arsenic removal, which indicates that iron-activated BC has high absorption capacity. In the MTW reaction, high sulfate activity promotes schwertmannite stability (Fig. 8), and the likelihood of its conversion to goethite or hematite is diminished. Competing ions may impact arsenic removal, and sulfate sorption to dairy waste BC has been shown to exhibit increased affinity with increased acidity (Zhao et al. 2019a). It is therefore expected that sulfate will compete with oxyanion arsenate for surface complexation on forest waste biochar in low-pH waters.

We propose that soluble arsenic was sequestered in the solid phase following an initial first step of ferrihydrite nucleation on BC followed by arsenic absorption during the first reaction hour. Ferrihydrite then transformed to goethite at pH 4 and room temperature, where the rate of ferrihydrite transformation is driven by dissolution and reprecipitation at pH 3–5 (Cudennec and Lecerf 2006; Das et al. 2013). The meta-stability of schwertmannite at the end of the experiment is attributed to the rate of transformation to goethite being inhibited by high aqueous sulfate activity, low pH, and ambient temperature (Jönsson et al. 2005). The thermodynamically predicted stable iron phases, with respect to pH and redox (Eh, volts) under the environmental conditions of the kinetic experiment, included goethite under oxidizing conditions across a wide pH range (2 to 12) and Fe^2+^_*(aq)*_ under suboxic conditions, and at very low pH (< 2.0), Fe^3+^_*(aq)*_ is stable (Fig. 8). In the absence of BC, iron suspensions or sediments in MTW were not produced within 48 h and are not thermodynamically favorable; it is therefore expected that surface catalysis on BC was necessary for ferric solid nucleation and arsenic removal.

## Conclusions

Biochar from pine forest waste produced at 450 °C proved to be effective at removing dissolved arsenic from AMD. This is the first work examining the substantial potential to employ unamended BC as an effective amendment for remediating mine drainage and tailing waters when both dissolved arsenate and ferric iron exist simultaneously. It was observed that the alkalinity of BC induced an increase of MTW pH, which caused dissolved ferric iron to become insoluble. As iron oxides precipitated at BC surfaces, goethite, ferrihydrite, and schwertmannite formed sorbate surface sites for arsenic removal. Arsenic adsorption kinetics were well described by a pseudo-second-order kinetic rate expression, consistent with chemisorption as the mechanism of arsenic removal from the aqueous phase. This investigation demonstrates that biochar is an effective remediation material for arsenic in AMD that is enriched in dissolved iron, and its use as a reactive barrier should be explored. Longer-term investigations are needed to examine the stability of arsenic absorbed by ferric oxide activated BC due to the meta-stability of the ferric solid phases.

## Supplementary Material

SuppMat

## Figures and Tables

**Table 1:** Inorganic solids in biochar before and after reaction with mine tailing water (MTW)

Sample	Inorganic solids
BC_t0_ (unrx)	Qz, Cal, Gr, Wh, Al
MTW-BC (h)	
12	Qz, Gr, Gp, Cal, 10 Å
24	Qz, Gr, Gp, Cal, 10 Å
48	Qz, Gr, Gp, Cal, Al, 10 Å
pH	
1.5	Qz, Gr, Gp, Al
2	Qz, Gr, Gp, Al
2.5	Qz, Gr, Gp, Al
3	Qz, Gr, Gp, Cal, Al
3.5	Qz, Gr, Gp, Cal, Al, 10 Å
4	Qz, Gr, Gp, Cal, Al, 10 Å
5	Qz, Gr, Gp, Cal, 10 Å
6	Qz, Gr, Gp, Cal, 10 Å
7	Qz, Gr, Gp. Cal
8	Qz, Gr, Gp, Cal, Al

Solid phases determined by synchrotron transmission X-ray diffraction. BC_t0_ (unrx) is unreacted pine waste biochar. Kinetic series and pH series samples were reacted with mine tailing water (MTW). Unreacted and time series XRD shown in Fig. 3, pH series XRD in Fig. S5; 10 Å aromatic is an undifferentiated peak characteristic of aromatic carbon rings. *Qz* quartz, *Cal* calcite, *Gr* graphite, *Gp* gypsum, *Al* albite, *Wh* whewellite

**Table 2:** Iron speciation of BC for 0–48 h from XANES linear combination fitting

BC sample (h)	Fit component (%)				*R*-factor
	BC	Fh	Sch	Goe	
0	100	–	–	–	NA
0.25	70	30	–	–	0.0348
1	60	24	–	16	0.0095
6	40	16	13	30	0.0119
48	30	23	15	32	0.0129

Sample 0, the unreacted biochar (BC at *t* = 0 min), was used as a fit component in reacted BC samples; the iron mineral fit components Fh = ferrihydrite, Sch = schwertmannite, and Goe = goethite were from Hayes et al. (2014). The *R*-factor represents the mean square misfit between the data and the fit. Component fits were normalized to unity

## References

[R1] Ahmad M, Lee SS, Lee SE, Al-Wabel MI, Tsang DCW, Ok YS (2016) Biochar-induced changes in soil properties affected immobilization/mobilization of metals/metalloids in contaminated soils. J Soils Sediments 17:717–730. 10.1007/s11368-015-1339-4

[R2] Amen R (2020) A critical review on arsenic removal from water using biochar-based sorbents: the significance of modification and redox reactions. Chem Eng J 396:125195. 10.1016/j.cej.2020.125195

[R3] Artiola JF, Rasmussen C, Freitas R (2012) Effects of a biochar-amended alkaline soil on the growth of romaine lettuce and bermudagrass. Soil Sci 177:561–570. 10.1097/SS.0b013e31826ba908

[R4] Asta MP, Cama J, Martínez M, Giménez J (2009) Arsenic removal by goethite and jarosite in acidic conditions and its environmental implications. J Hazard Mater 171:965–972. 10.1016/j.jhazmat.2009.06.097 19628332

[R5] ATSDR (2011) Substance Priority List. Agency for Toxic Substances and Disease Registry. http://www.atsdrcdc.gov/SPL/index.html

[R6] Bakshi S, Banik C, Rathke S, Laird D (2018) Arsenic sorption on zero-valent ironbiochar complexes. Water Res 137:153–163. 10.1016/j.watres.2018.03.021 29554531

[R7] Beaulieu BT, Savage KS (2005) Arsenate adsorption structures on aluminum oxide and phyllosilicate mineral surfaces in smelter-impacted soils. Environ Sci Technol 39:3571–3579. 10.1021/es048836f 15952360

[R8] Beesley L, Moreno-Jimenez E, Gomez-Eyles JL (2010) Effects of biochar and greenwaste compost amendments on mobility, bioavailability and toxicity of inorganic and organic contaminants in a multi-element polluted soil. Environ Pollut 158:2282–2287. 10.1016/j.envpol.2010.02.003 20219274

[R9] Beesley L, Marmiroli M, Pagano L, Pigoni V, Fellet G, Fresno T, Vamerali T, Bandiera M, Marmiroli N (2013) Biochar addition to an arsenic contaminated soil increases arsenic concentrations in the pore water but reduces uptake to tomato plants (Solanum lycopersicum L.). Sci Total Environ 454–455:598–603. 10.1016/j.scitotenv.2013.02.047 23583727

[R10] Bethke CM (2008) Geochemical and biogeochemical reaction modeling, 2nd edn. Cambridge University Press, Cambridge

[R11] Brennan JK, Bandosz TJ, Thomson KT, Gubbins KE (2001) Water in porous carbons. Colloids Surf A Physicochem Eng Asp 187-188: 539–568. 10.1016/S0927-7757(01)00644-6

[R12] Burton ED, Bush RT, Johnston SG, Watling KM, Hocking RK, Sullivan LA, Parker GK (2009) Sorption of arsenic(V) and arsenic(III) to schwertmannite. Environ Sci Technol 43:9202–9207. 10.1021/es902461x 19921855

[R13] Carlin DJ, Naujokas MF, Bradham KD, Cowden J, Heacock M, Henry HF, Lee JS, Thomas DJ, Thompson C, Tokar EJ, Waalkes MP, Birnbaum LS, Suk WA (2016) Arsenic and environmental health: state of the science and future research opportunities. Environ Health Perspect 124:890–899. 10.1289/ehp.1510209 26587579 PMC4937867

[R14] Cataldo S, Gianguzza A, Milea D, Muratore N, Pettignano A, Sammartano S (2018) A critical approach to the toxic metal ion removal by hazelnut and almond shells. Environ Sci Pollut Res 25:4238–4253. 10.1007/s11356-017-0779-3 29178014

[R15] Cudennec Y, Lecerf A (2006) The transformation of ferrihydrite into goethite or hematite, revisited. J Solid State Chem 179:716–722. 10.1016/j.jssc.2005.11.030

[R16] Das S, Jean J-S, Kar S (2013) Bioaccessibility and health risk assessment of arsenic in arsenic-enriched soils, Central India. Ecotoxicol Environ Saf 92:252–257. 10.1016/j.ecoenv.2013.02.016 23523002

[R17] Delany JM, Lundeen SR (1990) The LLNL thermochemical database. Lawrence Livermore National Laboratory Report UCRL-21658

[R18] Dixit S, Hering JG (2003) Comparison of arsenic(V) and arsenic(III) sorption onto iron oxide minerals: implications for arsenic mobility. Environ Sci Technol 37:4182–4189. 10.1021/es030309t 14524451

[R19] Farquhar ML, Charnock JM, Livens FR, Vaughan DJ (2002) Mechanisms of arsenic uptake from aqueous solution by interaction with goethite, lepidocrocite, mackinawite, and pyrite: an x-ray absorption spectroscopy study. Environ Sci Technol 36:1757–1762. 10.1021/es010216g 11993874

[R20] Forray FL, Smith AML, Navrotsky A, Wright K, Hudson-Edwards KA, Dubbin WE (2014) Synthesis, characterization and thermochemistry of synthetic Pb-As, Pb-Cu and Pb-Zn jarosites. Geochim Cosmochim Acta 127:107–119

[R21] Foster AL, Brown GE, Tingle TN, Parks GA (1998) Quantitative arsenic speciation in mine tailings using X-ray absorption spectroscopy. Am Mineral 83:553–568

[R22] Gao X, Root RA, Farrell J, Ela W, Chorover J (2013) Effect of silicic acid on arsenate and arsenite retention mechanisms on 6-L ferrihydrite: a spectroscopic and batch adsorption approach. Appl Geochem 38: 110–120 25382933 10.1016/j.apgeochem.2013.09.005 PMC4223807

[R23] Grossl PR, Eick M, Sparks DL, Goldberg S, Ainsworth CC (1997) Arsenate and chromate retention mechanisms on goethite. 2. Kinetic evaluation using a pressure-jump relaxation technique. Environ Sci Technol 31:321–326. 10.1021/es950654l

[R24] Hammond CM, Root RA, Maier RM, Chorover J (2018) Mechanisms of arsenic sequestration by Prosopis juliflora during the phytostabilization of metalliferous mine tailings. Environ Sci Technol 52:1156–1164. 10.1021/acs.est.7b04363 29241010 PMC5930015

[R25] Hammond CM, Root RA, Maier RM, Chorover J (2020) Arsenic and iron speciation and mobilization during phytostabilization of pyritic mine tailings. Geochim Cosmochim Acta 286:306–323. 10.1016/j.gca.2020.07.001 33071297 PMC7556726

[R26] Hayes SM, Root RA, Perdrial N, Maier RM, Chorover J (2014) Surficial weathering of iron sulfide mine tailings under semi-arid climate. Geochim Cosmochim Acta 141:240–257. 10.1016/j.gca.2014.05.030 25197102 PMC4151187

[R27] Helgeson HC, Kirkham DH, Flowers GC (1981) Theoretical prediction of the thermodynamic behaviour of aqueous electrolytes at high pressures and temperatures. IV. Calculation of activities coefficients, osmotic coefficients, and apparent molal and standard and relative partial molal properties to 5 kb and 600 °C. Am J Sci 281:1241–1516

[R28] Hu B, Song Y, Wu S, Zhu Y, Sheng G (2019) Slow released nutrient-immobilized biochar: a novel permeable reactive barrier filler for Cr(VI) removal. J Mol Liq 286:110876. 10.1016/j.molliq.2019.04.153

[R29] Ibrahim M, Khan S, Hao X, Li G (2016) Biochar effects on metal bioaccumulation and arsenic speciation in alfalfa (Medicago sativa L.) grown in contaminated soil. Int J Environ Sci Technol 13:2467–2474. 10.1007/s13762-016-1081-5

[R30] ICDD (2005) JCPDS Powder Diffraction File 2 Database. International Centre for Diffraction Data, Newton Square, PA, USA

[R31] Ilavsky J (2012) Nika: software for two-dimensional data reduction. J Appl Crystallogr 45:324–328. 10.1107/s0021889812004037

[R32] Jin Q (2020) Grape pomace and its secondary waste management: biochar production for a broad range of lead (Pb) removal from water. Environ Res 186:109442. 10.1016/j.envres.2020.109442 32302873

[R33] Jönsson J, Persson P, Sjoberg S, Lovgren L (2005) Schwertmannite precipitated from acid mine drainage: phase transformation, sulphate release and surface properties. Appl Geochem 20:179–191

[R34] Kappler A, Wuestner ML, Ruecker A, Harter J, Halama M, Behrens S (2014) Biochar as an electron shuttle between bacteria and Fe(III) minerals. Environ Sci Technol Lett 1:339–344. 10.1021/ez5002209

[R35] Kelly CN, Peltz CD, Stanton M, Rutherford DW, Rostad CE (2014) Biochar application to hardrock mine tailings: soil quality, microbial activity, and toxic element sorption. Appl Geochem 43:35–48. 10.1016/j.apgeochem.2014.02.003

[R36] Klüpfel L, Keiluweit M, Kleber M, Sander M (2014) Redox properties of plant biomass-derived black carbon (biochar). Environ Sci Technol 48:5601–5611. 10.1021/es500906d 24749810

[R37] Lehmann J, Joseph S (eds) (2009) Biochar for environmental management: science and technology. Earthscan, London & Sterling

[R38] Lehmann J, Liang B, Solomon D, Lerotic M, Luizão F, Kinyangi J, Schäfer T, Wirick S, Jacobsen C (2005) Near-edge X-ray absorption fine structure (NEXAFS) spectroscopy for mapping nano-scale distribution of organic carbon forms in soil: application to black carbon particles. Glob Biogeochem Cycles 19. 10.1029/2004gb002435

[R39] Lerotic M, Mak R, Wirick S, Meirer F, Jacobsen C (2014) MANTiS: a program for the analysis of X-ray spectromicroscopy data. J Synchrotron Radiat 21:1206–1212. 10.1107/S1600577514013964 25178014

[R40] Li F, Li Z, Mao P, Li Y, Li Y, McBride MB, Wu J, Zhuang P (2018) Heavy metal availability, bioaccessibility, and leachability in contaminated soil: effects of pig manure and earthworms. Environ Sci Pollut Res Int 26:20030–20039. 10.1007/s11356-018-2080-5 29705900

[R41] Li X, Zhang X, Wang X, Cui Z (2019) Phytoremediation of multi-metal contaminated mine tailings with Solanum nigrum L. and biochar/attapulgite amendments. Ecotoxicol Environ Saf 180:517–525. 10.1016/j.ecoenv.2019.05.033 31128549

[R42] Liang C, Gascó G, Fu S, Méndez A, Paz-Ferreiro J (2016) Biochar from pruning residues as a soil amendment: effects of pyrolysis temperature and particle size. Soil Tillage Res 164:3–10. 10.1016/j.still.2015.10.002

[R43] Liu X (2019) Impact of biochar amendment on the abundance and structure of diazotrophic community in an alkaline soil. Sci Total Environ 688:944–951. 10.1016/j.scitotenv.2019.06.293 31726576

[R44] Lu K, Yang X, Gielen G, Bolan N, Ok YS, Niazi NK, Xu S, Yuan G, Chen X, Zhang X, Liu D, Song Z, Liu X, Wang H (2016) Effect of bamboo and rice straw biochars on the mobility and redistribution of heavy metals (Cd, Cu, Pb and Zn) in contaminated soil. J Environ Manag 186:285–292. 10.1016/j.jenvman.2016.05.068 27264699

[R45] Maillot F, Morin G, Juillot F, Bruneel O, Casiot C, Ona-Nguema G, Wang Y, Lebrun S, Aubry E, Vlaic G, Brown GE Jr (2013) Structure and reactivity of As(III)- and As(V)-rich schwertmannites and amorphous ferric arsenate sulfate from the Carnoulès acid mine drainage, France: comparison with biotic and abiotic model compounds and implications for As remediation. Geochim Cosmochim Acta 104:310–329

[R46] Majzlan J, Navrotsky A, McCleskey RB, Alpers CN (2006) Thermodynamic properties and crystal structure refinement of ferricopiapite, coquimbite, rhomboclase, and Fe_2_(SO_4_)_3_(H_2_O)_5_. Eur J Mineral 18:175–186. 10.1127/0935-1221/2006/0018-0175

[R47] Manning BA, Fendorf SE, Bostick BC, Susarez D (2002) Arsenic(III) oxidation and arsenic(V) adsorption reactions on synthetic birnessite. Environ Sci Technol 36(5):976–981 11918029 10.1021/es0110170

[R48] Mendez MO, Maier RM (2008) Phytostabilization of mine tailings in arid and semiarid environments - an emerging remediation technology. Environ Health Perspect 116:278–283 18335091 10.1289/ehp.10608 PMC2265025

[R49] Meng J, Tao M, Wang L, Liu X, Xu J (2018) Changes in heavy metal bioavailability and speciation from a Pb-Zn mining soil amended with biochars from co-pyrolysis of rice straw and swine manure. Sci Total Environ 633:300–307. 10.1016/j.scitotenv.2018.03.199 29574374

[R50] Ona-Nguema G, Morin G, Juillot F, Calas G, Brown GE (2005) EXAFS analysis of arsenite adsorption onto two-line ferrihydrite, hematite, goethite, and lepidocrocite. Environ Sci Technol 39:9147–9155. 10.1021/es050889p 16382936

[R51] Paktunc D, Dutrizac JE (2003) Characterization of arsenate-for-sulfate substitution in synthetic jarosite using X-ray diffraction and X-ray absorption spectroscopy. Can Mineral 41:905–919. 10.2113/gscanmin.41.4.905

[R52] Puga AP, Melo LCA, de Abreu CA, Coscione AR, Paz-Ferreiro J (2016) Leaching and fractionation of heavy metals in mining soils amended with biochar. Soil Tillage Res 164:25–33. 10.1016/j.still.2016.01.008

[R53] Ravel B, Newville M (2005) ATHENA, ARTEMIS, HEPHAESTUS: data analysis for X-ray absorption spectroscopy using IFEFFIT. J Synchrotron Radiat 12:537–541 15968136 10.1107/S0909049505012719

[R54] Rehr JJ (1993) Recent developments in multiple-scattering calculations of XAFS and XANES Japan. J Appl Phys 32:8–12

[R55] Root RA, Dixit S, Campbell KM, Jew AD, Hering JG, O’Day PA (2007) Arsenic sequestration by sorption processes in high-iron sediments. Geochim Cosmochim Acta 71:5782–5803

[R56] Root RA, Hayes SM, Hammond CM, Maier RM, Chorover J (2015) Toxic metal(loid) speciation during weathering of iron sulfide mine tailings under semi-arid climate. Appl Geochem 62:131–149. 10.1016/j.apgeochem.2015.01.005 26549929 PMC4632981

[R57] Savage KS, Tingle TN, O’Day PA, Waychunas GA, Bird DK (2000) Arsenic speciation in pyrite and secondary weathering phases, Mother Lode Gold District, Tuolumne County, California. Appl Geochem 15:1219–1244

[R58] Sherman D, Randal SR (2003) Surface complexation of arsenic(V) to iron(III) (hydr)oxides: structural mechanism from ab initio molecular geometries and EXAFS spectroscopy. Geochim Cosmochim Acta 67:4223–4230

[R59] Solis-Dominguez FA, White SA, Hutter TB, Amistadi MK, Root RA, Chorover J, Maier RM (2012) Response of key soil parameters during compost-assisted phytostabilization in extremely acidic tailings: effect of plant species. Environ Sci Technol 46:1019–1027. 10.1021/es202846n 22191663 PMC3263829

[R60] Solomon D (2012) Micro- and nano-environments of carbon sequestration: multi-element STXM–NEXAFS spectromicroscopy assessment of microbial carbon and mineral associations. Chem Geol 329: 53–73. 10.1016/j.chemgeo.2012.02.002

[R61] Stookey L (1970) Ferrozine—a new spectrophotometric reagent for iron. Anal Chem 42(7):779–781. 10.1021/ac60289a016

[R62] Uchimiya M, Bannon DI, Wartelle LH (2012) Retention of heavy metals by carboxyl functional groups of biochars in small arms range soil. J Agric Food Chem 60:1798–1809. 10.1021/jf2047898 22280497

[R63] Valentin-Vargas A, Chorover J, Maier RM (2013) A new standard-based polynomial interpolation (SBPIn) method to address gel-to-gel variability for the comparison of multiple denaturing gradient gel electrophoresis profile matrices. J Microbiol Methods 92:173–177. 10.1016/j.mimet.2012.12.001 23234884 PMC3570647

[R64] Voegelin A, Weber F-A, Kretzschmar R (2007) Distribution and speciation of arsenic around roots in a contaminated riparian floodplain soil: micro-XRF element mapping and EXAFS spectroscopy. Geochim Cosmochim Acta 71:5804–5820

[R65] Wang S, Gao B, Zimmerman AR, Li Y, Ma L, Harris WG, Migliaccio KW (2015) Removal of arsenic by magnetic biochar prepared from pinewood and natural hematite. Bioresour Technol 175:391–395. 10.1016/j.biortech.2014.10.104 25459847

[R66] Webb SM (2006) SixPACK: a graphical user interface for XAS analysis using IFEFFIT. Phys Scr T 115:1011–1014

[R67] WHO (2017) Guidelines for drinking-water quality, 4th edn. World Health Organization, Geneva ISBN 978-92-4-154995-0 28759192

[R68] Wilkie JA, Hering JG (1996) Adsorption of arsenic onto hydrous ferric oxide: effects of adsorbate/adsorbent ratios and co-occurring solutes. Colloids Surf A Physicochem Eng Asp 107:97–110. 10.1016/0927-7757(95)03368-8

[R69] Wu C, Cui M, Xue S, Li W, Huang L, Jiang X, Qian Z (2018) Remediation of arsenic-contaminated paddy soil by iron-modified biochar. Environ Sci Pollut Res Int 25:20792–20801. 10.1007/s11356-018-2268-8 29756185

[R70] Xiao X, Chen B, Chen Z, Zhu L, Schnoor JL (2018) Insight into multiple and multilevel structures of biochars and their potential environmental applications: a critical review. Environ Sci Technol 52:5027–5047. 10.1021/acs.est.7b06487 29634904 PMC6402350

[R71] Yee N, Shaw S, Benning L, Nguyen TH (2006) The rate of ferrihydrite transformation to goethite via the Fe(II) pathway. Am Mineral 91: 92–96. 10.2138/am.2006.1860

[R72] Yoon K, Cho D-W, Tsang DCW, Bolan N, Rinklebe J, Song H (2017) Fabrication of engineered biochar from paper mill sludge and its application into removal of arsenic and cadmium in acidic water. Bioresour Technol 246:69–75. 10.1016/j.biortech.2017.07.020 28712779

[R73] Yu L, Yuang Y, Tang J, Wang Y, Zhou S (2015) Biochar as an electron shuttle for reductive dechlorination of pentachlorophenol by Geobacter sulfurreducens. Sci Rep 5:16221–16221. 10.1038/srep16221 26592958 PMC4655402

[R74] Yuan J-H, Xu R-K, Zhang H (2011) The forms of alkalis in the biochar produced from crop residues at different temperatures. Bioresour Technol 102:3488–3497. 10.1016/j.biortech.2010.11.018 21112777

[R75] Zhang M, Gao B, Varnoosfaderani S, Hebard A, Yao Y, Inyang M (2013) Preparation and characterization of a novel magnetic biochar for arsenic removal. Bioresour Technol 130(1):457–462. 10.1016/j.biortech.2012.11.132 23313693

[R76] Zhao B, Xu H, Ma F, Zhang T, Nan X (2019a) Effects of dairy manure biochar on adsorption of sulfate onto light sierozem and its mechanisms. RSC Adv 9:5218–5223. 10.1039/C8RA08916G PMC9060684 35514648

[R77] Zhao J, Shen X-J, Domene X, Alcañiz J-M, Liao X, Palet C (2019b) Comparison of biochars derived from different types of feedstock and their potential for heavy metal removal in multiple-metal solutions. Sci Rep 9:9869. 10.1038/s41598-019-46234-4 31285499 PMC6614460

