# Supplementary material for: Biochar-templated surface precipitation and inner-sphere complexation effectively removes arsenic from acid mine drainage
Source: Environ Sci Pollut Res Int. Author manuscript; Available in PMC 2021 Sep 1. (PMC8364533; doi:10.1007/s11356-021-13869-8)
Supplement: SuppMat [file NIHMS1698084-supplement-SuppMat.docx]

Supplementary Information

**Biochar-templated surface precipitation and inner-sphere complexation removes arsenic from acid mine drainage**

Dongmei Wang^1,2^, Robert A. Root^1^, and Jon Chorover^1*^

^1^ Department of Environmental Science, University of Arizona, Tucson, AZ 85721

^2^ Department of Environmental Engineering, Southwest Jiaotong University, Chengdu, 610031, China

^*^Corresponding author: J Chorover,

Department of Environmental Science, University of Arizona, 1177 E 4^th^ St, Shantz 429, Tucson, AZ 85721

telephone: +1 520-626-5635, fax: 520-626-1647, e-mail: [chorover@arizona.edu](mailto:chorover@arizona.edu).

**Pages: 12**

**Figures**

Fig. S1 Buffering of BC with pH adjustments

Fig. S2 Effects of pH on arsenic removal with and without biochar

Fig. S3 Arsenic adsorption kinetic models

Fig. S4 FTIR spectrum of schwertmannite

Fig. S5 XRD with pH 1.5-8

Fig. S6 Carbon NEXAFS of biochar DOM

Fig. S7 As XANES and EXAFS LCF

Fig. S8 Arsenic reacted with and without BC in the absence of MTW

Fig. S9 Relationship of the removal of As and Fe from mine water.

**Tables**

S1 Physicochemical properties of mine tailings water and mine tailings

S2 Properties of pine forest waste biochar

S3 Thermodynamic data for conditions in geochemical Eh-pH diagrams

S4 As EXAFS fits for MTW-BC 48 h

**Table S1.** Physiochemical characterization of mine tailings water (MTW) and mine tailings.

| Mine tailings water^a^ (mg kg^-1^) | | |  |  |  |  |  |
| --- | --- | --- | --- | --- | --- | --- | --- |
| pH | DIC | DOC | EC | S dissolved | Fe^2+^ | As^5+^/As_total_ |  |
|  | mg kg^-1^ | mg kg^-1^ | mS cm^-1^ | mg kg^-1^ | mg kg^-1^ |  |  |
| 3.2(0.1) | 48.7(1.1) | 59.9(1.4) | 7.22(0.01) | 7260(115) | 85.8(1.9) | 1.0 |  |
| Al | Cr | Mn | Fe | Cu | Zn | As | Pb |
| 1360(110) | 0.37(0.01) | 56.9(0.2) | 1340(50) | 58.9(3.0) | 1500(60) | 0.58(0.01) | 0.26(0.01) |
| Tailings elemental analysis ^b^ (mg kg^-1^) | | | |  |  |  |  |
| Al | Cr | Mn | Fe | Cu | Zn | As | Pb |
| 3250(300) | 6.84(0.44) | 79.6(4.5) | 45100(3200) | 79.0(6) | 1540 (90) | 1950(110) | 1390(120) |

Mine tailings from Iron King Mine-Humboldt Smelter Site (IKMHSS) Superfund Site, located in central Arizona, USA. Results given as mean and (standard deviation, n=3). DIC = dissolved inorganic carbon, DOC = dissolved organic carbon, EC = electrical conductivity, ^a^ released form water soluble fraction in 1:10 extraction with 18.2 MΩ DI water, reported as mg of analyte kg^-1^ of solution, ^b^ micro-wave assisted digestion reported as mg of analyte kg^-1^ of tailings.

**Table S2.** Properties of pine forest waste biochar.

| Particle density^a^ | Porosity^a^ | pH | DIC | DOC | EC |
| --- | --- | --- | --- | --- | --- |
| g cm^-3^ | % |  | mg kg^-1^ | mg kg^-1^ | mS cm^-1^ |
| 1.51^a^ | 86 | 10.0(0.1) | 181.4(2.2) | 234.3(0.6) | 0.76(0.02) |
| Total elemental analysis^b^ (mg kg^-1^) | | | | | |
| Na | Mg | K | Ca | P | S |
| 240(20) | 769(30) | 2408(83) | 6179(475) | 495 | 186 |
| Al | Mn | Fe | Zn | As | Pb |
| 1084(142) | 257(4) | 821(33) | 19.9(6.9) | 0.60(0.40) | 1.83(0.26) |
| Water soluble^c^- (mg kg^-1^) | | | | | |
| Na | Mg | K | Ca | PO_4_^3-^ | SO_4_^2-^ |
| 19.7(3.7)c | 123(5) | 666(20) | 161(6) | 0.66 (0.04) | 29.4^c^ |
| Al | Mn | Fe | Zn | As | Pb |
| 8.13(0.43) | 0.20(0.2) | 0.006 | 0.60(0.1) | 0.0056 | 0.04(0.01) |

^a^ ([Artiola et al. 2012](#_ENREF_1)), ^b^ microwave assisted HNO_3_ digestion, ^c^ water soluble in 18.2 MΩ milli-Q water, reported as mg of analyte kg^-1^ of pine forest biochar.


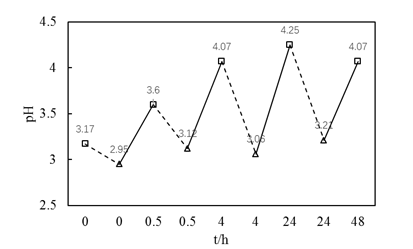
**Fig. S1** Proton buffering ability of BC. Dash line refers to pH adjustment with acid. Solid line refers to pH increase with BC.


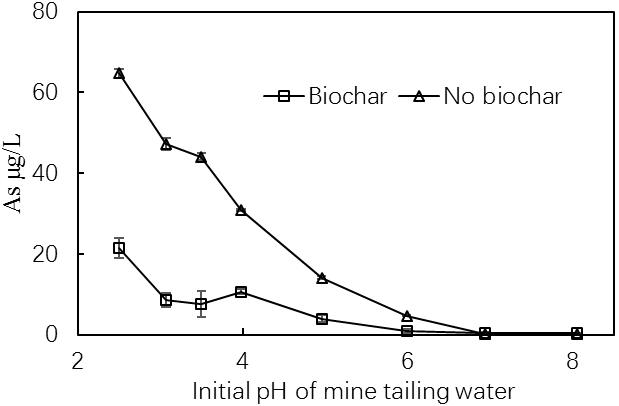


**Figure S2.** Effects of initial and adjusted pH in MTW on arsenic removal with and without biochar. Error bars are generally smaller than the symbols and represent standard deviation of triplicate measurements.


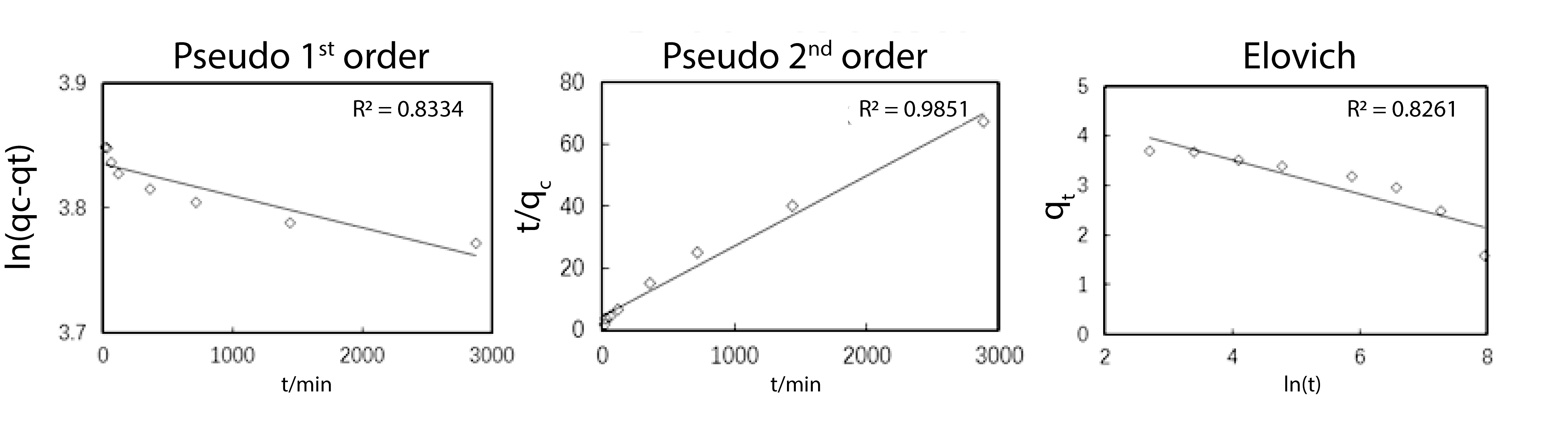


**Fig. S3** Arsenic adsorption fit to kinetic models ([Jin et al. 2020](#_ENREF_3); [Özacar and Şengil 2005](#_ENREF_6)).

**S1. Effect of pH on BC arsenic removal**

We studied the buffering ability of BC by controlling the reaction solution pH by adding acid (**Fig. S1**). The pH of the solution was initially around 3. Squares and solid lines represent pH value being increased by BC and triangles with dashed lines indicate pH being decreased by adjustment with acid. The pH rebound is almost the same at each time step despite adding acid each time. The pH of the mine tailings water was 2.4. Experimental results demonstrate BC’s buffering capacity within this acid range.

To test the effect of pH on the removal of arsenic, reactions were carried out at pH between 2.5 and 8 (**Fig. S2**). Removal of arsenic form solution as a function of pH values at different reaction time are shown. Whereas arsenic removal was greatest for the samples with BC, at higher pH (e.g. pH ≥ 6) arsenic partitioned to the solid phase even in the absence of BC. A decrease in aqueous phase arsenic was observed from pH 2.5 to 5 for the control and BC samples; the difference between aqueous arsenic in the BC-free and BC added solutions represents the capacity of arsenic removal by BC alone. It is observed that pH plays an important role for arsenic removal, but the addition of BC results in a >3x decrease in soluble arsenic at lower pH.

**S2. Arsenic sorption kinetics**

The removal of arsenic form mine water was investigated over timescales from 15 minutes to almost 3000 minutes. The portioning of arsenic from the aqueous phase onto the solid iron activated biochar was modeled with appropriate rate expressions (Fig S3). The rate of reaction was best fit with a pseudo 2^nd^ order rate law, and the sorption mechanism is classified as pseudo due to the requirement of the precursor precipitation of iron oxides at the BC surface to catalyze the sorption of arsenic.

**
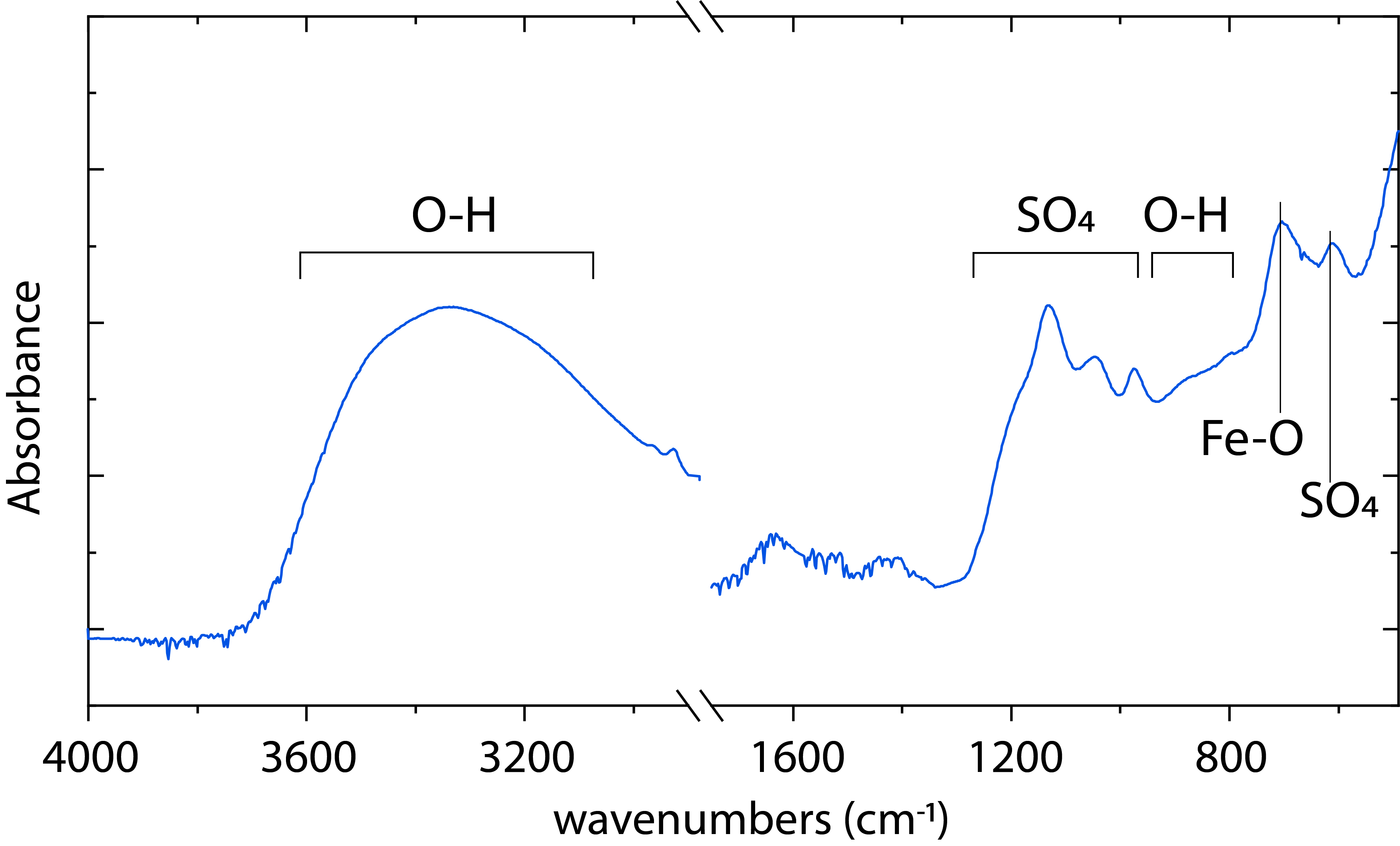
**

**Fig. S4** FTIR spectra schwertmannite showing characteristic SO_4_ bands at 950-1200 cm^-1^.

**
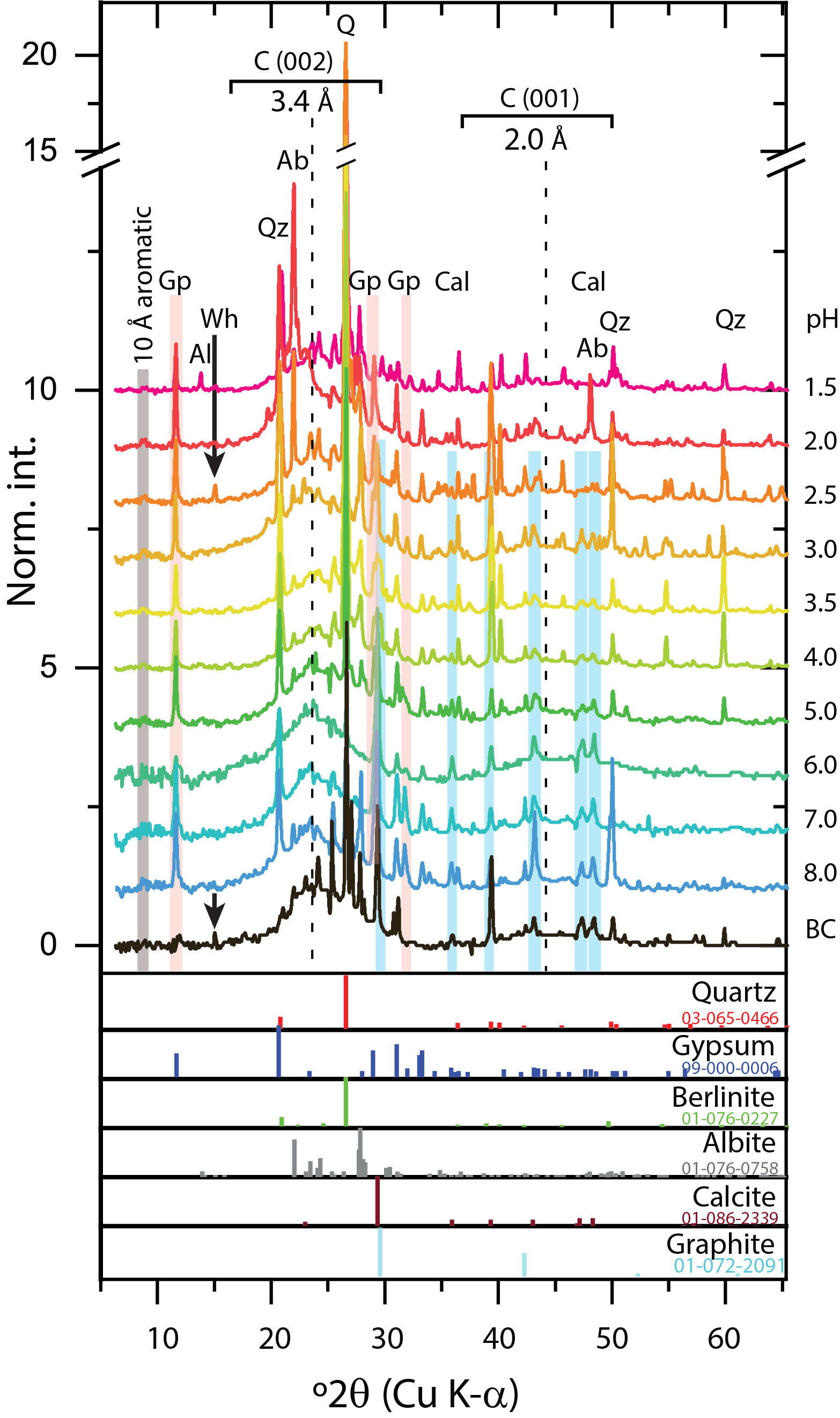
**

**Fig. S5** The X-ray diffraction for the unreacted BC and MTW-BC at pH form 1.5 to 8. Broad features are noted at 3.4Å (normalization peak) and 2.0 Å that are indicative of turbostratic graphitic C. Cal= calcite (noted by blue bands) , Qz = quartz, Gp = gypsum (noted by pink bands), Gr = graphite, Wh = whewellite, 10 Å aromatic (noted in gray band) is an undifferentiated peak characteristic of aromatic C rings.

**
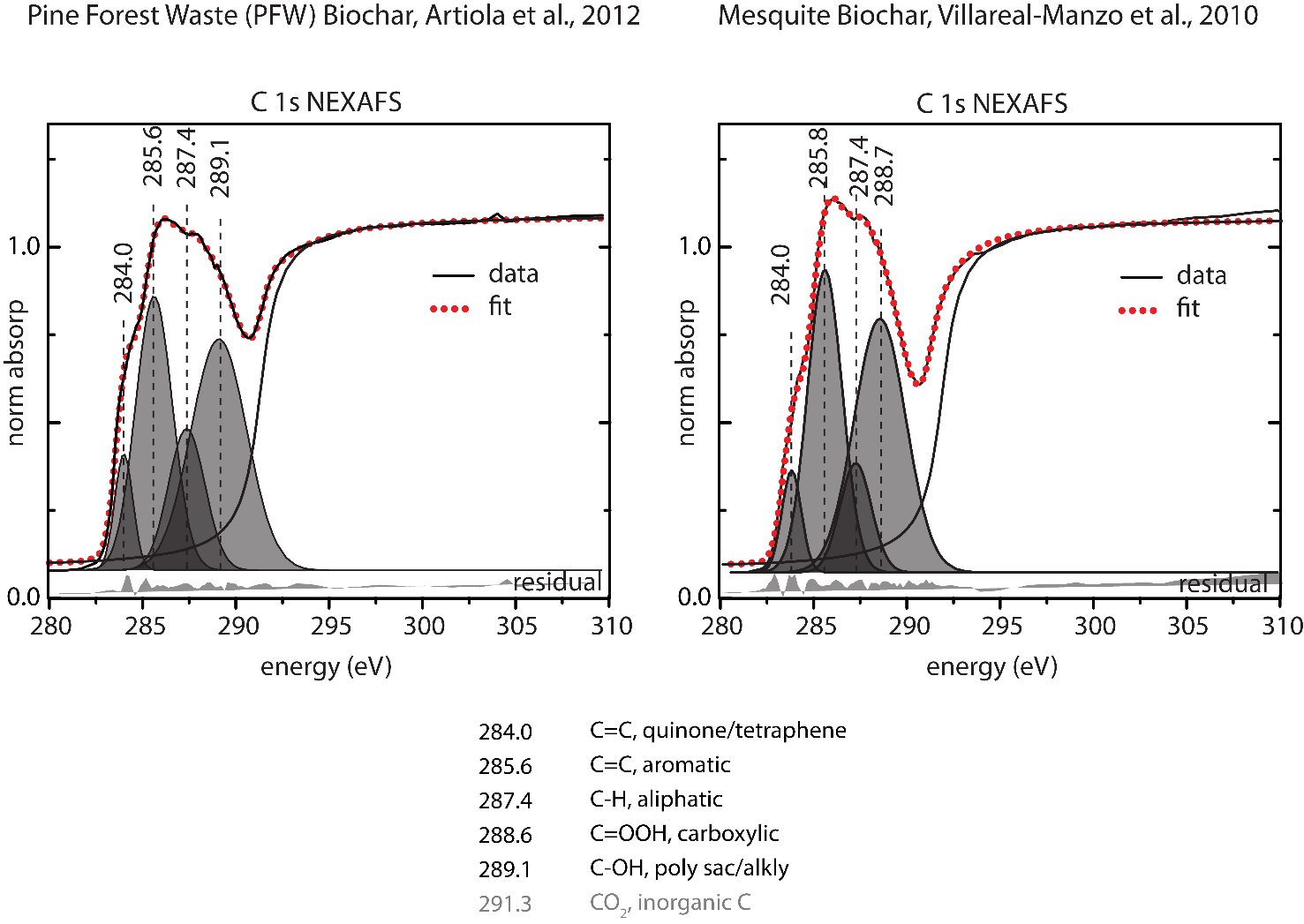
**The BC DOM (<1.2 µm) sample characterized by C NEXAFS lacked a dominant large peak at 285 eV, indicating relatively low graphitic content (**Fig. S6**). The peaks at 284 and 285.6 eV indicate C=C quinone and aromatic C structures, respectively. The smaller peak at 287.4 eV indicates aliphatic-C, long-range ordered C, and is consistent with pyrolysis temperatures ~450^o^C. The BC DOM had stronger contributions from aliphatic C compared to graphitic C.

**Fig. S6** Carbon NEXAFS of dissolved organic carbon derived from pine forest waste biochar. Peak features at 284.0 eV attributed to a quinone-C surface group 1s-π*(C=O); 285.6 eV from aromatic-C ring 1s-π*(C=C); 287.4 eV aliphatic-C 1s-σ*(3p_C-H_); and 289.1 eV generally assigned to O-alkyl-C and polysaccharide-C 1s-σ*(3p_C-OH_) and 1s-π*(C=O).


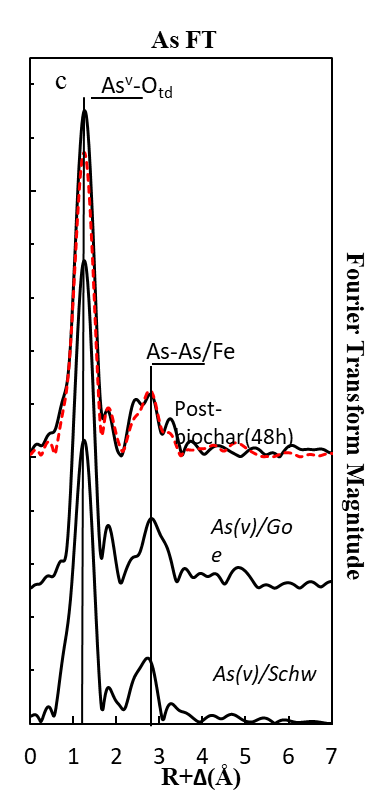

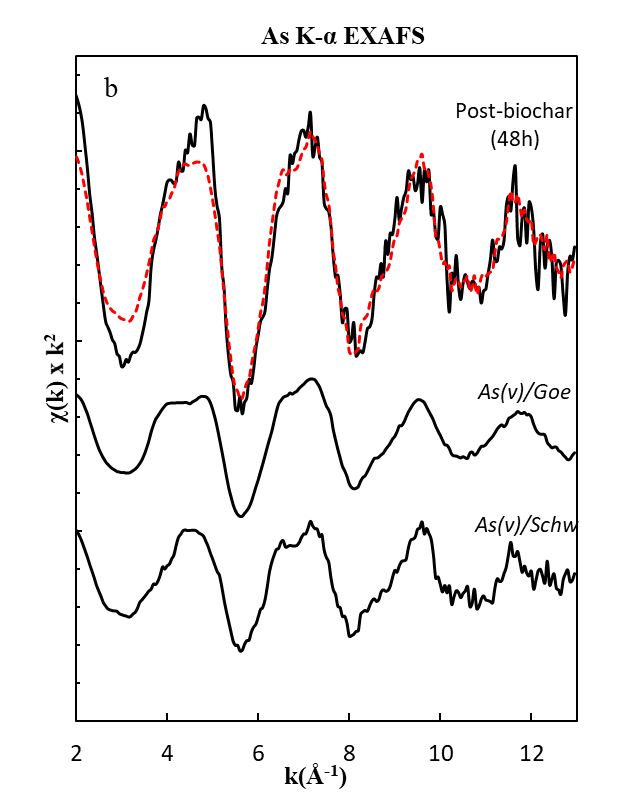

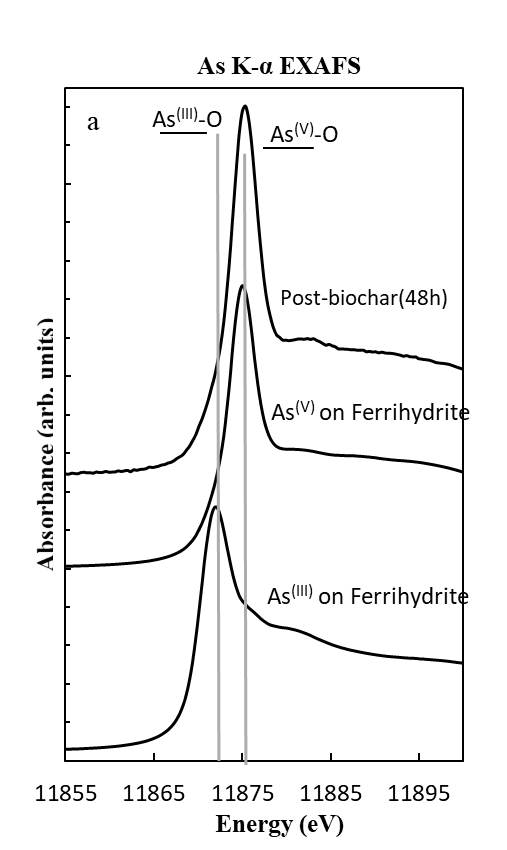


**Fig. S7** Arsenic K-edge XANES spectra on MTW-BC(48h). Reacted biochar shows only As^(v)^. Arsenic k^2^ EXAFS examined by linear combination fitting (LCF) show good correlation (R-factor 0.1089) with a combination of As^(v)^ sorbed goethite and As^(v)^ coprecipitated with schwertmannite. LCF has limited applicability is quantitative deconvolution of sorbed species on ferric hydroxy(sulf)oxides.


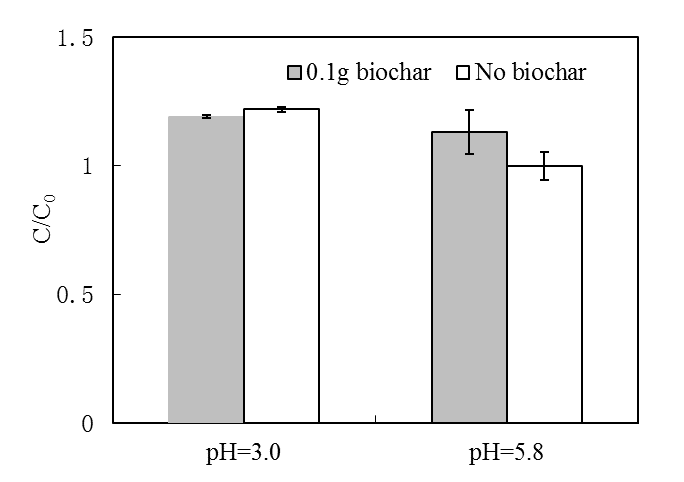
**Fig. S8** Result of dissolved As^(V)^ reacted with and without biochar in the absence of mine tailings water. at pH 3.0 and 5.8. The arsenic solution was prepared with HAsNa_2_O_4_·7H_2_O and adjusted to pH = 3.0 and 5.8 with 1M hydrochloric acid.


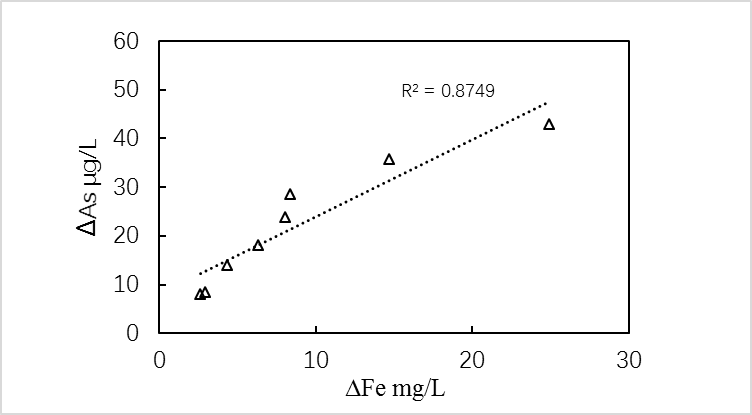


**Fig. S9** The relationship of arsenic and iron removal from mine tailings water. ∆As and ∆Fe represent the difference in concentrations of arsenic and iron in mine tailings water between blank and biochar reacted experiments, respectively at the kinetic intervals examined. Fe:As linear relation of 525 shown as dashed line.

**Table S3:** Thermodynamic data for conditions in geochemical Eh-pH diagrams

log *K_sp_*

*Iron solid*

Ferrihydrite [Fe(OH)_3_]+ 3H^+^ 🡘 Fe^3+^ + H_2_O 3.0^a^

Jarosite [KFe_3_(SO_4_)_2_(OH)_6_] + 6H^+^ 🡘 3Fe^3+^ + 2SO_4_^2-^ + K^+^ + 6H_2_O -9.37^b^

Pb-Jarosite [PbFe_6_(SO_4_)_4_(OH)_12_]+ 12H^+^ 🡘 6Fe^3+^ + 4SO_4_^2-^ + Pb^+^ + 12H_2_O -22.84^b^

Schwertmannite [Fe_8_O_8_(OH)_4.8_(SO_4_)_1.6_] + 20.8H^+^ 🡘 8Fe^3+^ + 12.8H_2_O + 1.6SO_4_^2-^ -17.4^c^

^a^ ([Majzlan et al. 2004](#_ENREF_5)); ^b^ ([Kashkay et al. 1975](#_ENREF_4)); ^c^ ([Bigham et al. 1996](#_ENREF_2)).

**Table S4.** Arsenic K-edge EXAFS fit for mine tailing water reacted biochar.

|  | As-L | | N | R  (Å) | | | σ^2^  (Å^2^) | ΔE_0_  (eV) | R-factor | red. χ | Sym^b^ | |
| --- | --- | --- | --- | --- | --- | --- | --- | --- | --- | --- | --- | --- |
| MTW-BC  48 h | | O | 4.0 | | 1.70 | 0.0018 | | -4.05 | 0.0201 | 116 | T_d_ |  |
|  |  | MS^c^ | 4.0^d^ | | 3.05 | 0.0024^d^ | |  |  |  | As-O-O-As |  |
|  |  | MS^c^ | 10.6^d^ | | 3.09 | 0.0024^d^ | |  |  |  | As-O-O-As |  |
|  | | Fe | 1.2 | | 3.37 | 0.0035 | |  |  |  | ^2^C |  |

N is the number of backscattering atoms at distance (R); σ^2^, the Debye–Waller term, is the absorber-backscatterer mean-square relative displacement; ΔE_0_ is the threshold energy difference; reduced chi (red. χ) and R-factor are goodness-of-fit parameters. Scale factor (S^2^_0_) = 1. ^b^ Sym –symmetry or coordination of backscattering. ^c^ Fit with a multiple scattering path from As–O–O–As in arsenate tetrahedra. ^d^ Parameter linked in fit to the parameter directly above.

Based on XANES showing only arsenate, the structure of arsenic in tetrahedral coordination to four apical oxygen atoms was used to constrain the coordination number (N) in EXAFS analysis by starting with a As-O = 4 and allowing σ^2^ to adjust in the fit. For the multiple scattering (MS) paths As-O-O-As within the arsenate tetrahedra, N was likewise fixed at 4 and 12 and based on path geometry and σ^2^_MS_ was linked to the adjusted σ^2^_As-O_ term for the As-O scattering calculation (4/3 σ^2^_As-O_). To achieve a good fit to the second shell, the As-Fe distance was allowed to vary, and converged in iterative fits at 3.37 Å, a distance consistent with ^2^C coordination of bidentate binuclear ligation to two non-edge sharing Fe (bridging) octahedra; however the coordination was lower than would be expected in ^2^C coordination at 1.2.

References

Artiola JF, Rasmussen C, Freitas R (2012) Effects of a Biochar-Amended Alkaline Soil on the Growth of Romaine Lettuce and Bermudagrass Soil Science 177:561-570 doi:10.1097/SS.0b013e31826ba908

Bigham JM, Schwertmann U, Traina SJ, Winland RL, Wolf M (1996) Schwertmannite and the chemical modeling of iron in acid sulfate waters Geochimica et Cosmochimica Acta 60:2111-2121

Jin Q et al. (2020) Grape pomace and its secondary waste management: Biochar production for a broad range of lead (Pb) removal from water Environmental Research 186:109442 doi:<https://doi.org/10.1016/j.envres.2020.109442>

Kashkay CM, Borovskaya YB, Babazade MA (1975) Determination of deltaGf 298 of synthetic jarosite and its sulfate analogues Geokhimiya 5:778-784

Majzlan J, Navrotsky A, Schwertmann U (2004) Thermodynamics of iron oxides. III - Enthalpies of formation oand stability of ferrihydrite (Fe(OH)3), schwertmannite (FeO(OH)3/4(SO4)i/8), and Fe2O3 Geochimica et Cosmochimica Acta 68:1049-1059

Özacar M, Şengil İA (2005) Adsorption of metal complex dyes from aqueous solutions by pine sawdust Bioresource Technology 96:791-795 doi:<https://doi.org/10.1016/j.biortech.2004.07.011>
